# A second update to the checklist of Finnish long-legged flies (Diptera: Dolichopodidae), with a re-evaluation of the status of *Hydrophorus callosoma* Frey, 1915

**DOI:** 10.3897/BDJ.1.e976

**Published:** 2013-10-28

**Authors:** Jere Kahanpää

**Affiliations:** † University of Helsinki, Helsinki, Finland

**Keywords:** Diptera, Dolichopodidae, *Hydrophorus*, Finland, new synonym, lectotype designation, faunistics

## Abstract

Eighteen species of long-legged flies (Dolichopodidae) are reported as new to Finland. A further species, *Microphorella praecox* (Loew), is confirmed as a Finnish species. The status of *Hydrophorus callosoma* Frey, 1915 is re-evaluated and a lectotype is designated for the species. *Hydrophorus albosignatus* Ringdahl, 1919 is found to be a junior synonym of *Hydrophorus callosoma* (**syn. n.**). Characters for identifying both sexes of *Hydrophorus callosoma* and *Hydrophorus altivagus* Aldrich are presented and illustrated with high-quality photographs.

## Introduction

The latest checklist of Finnish long-legged flies (Diptera: Dolichopodidae) was published nearly ten years ago (Kahanpää and Grichanov 2004). A single update paper was published soon afterwards (Kahanpää 2006). Additional dolichopodid species have since been reported from Finland by Kahanpää (2007), Kahanpää and Negrobov (2007) and Winqvist (2011).

This paper summarizes the changes that have occurred since the last update. A grand total of 18 species are added to the list of Finnish Dolichopodidae, bringing the total up to 264 species. The dolichopodid fauna of Finland is relatively well known, but at least 10-20 additional species can still be expected to occur in the country.

It should be noted that the former family Microphoridae, excluded from the previous national dolichopodid checklist but included in Kahanpää and Winqvist (2005), is now considered to be a subfamily of Dolichopodidae (Cumming and Brooks 2002).

## Materials and methods

The material examined is stored in the Finnish Museum of Natural History, University of Helsinki (MZH) and the author's private collection.

Stack photographs of *Hydrophorus* specimens were taken using a Canon EOS digital camera and a Canon MP-E 65 mm macro lens. To create a final photograph, 35-40 exposures of each subject were combined using the CombineZ program (Hadley 2010).

## Taxon treatments

### Microphorella praecox

(Loew, 1864)

http://www.faunaeur.org/full_results.php?id=129775

#### Materials

- **Occurrence:** recordedBy: Antti Haarto; individualCount: 2; sex: females; otherCatalogNumbers: labelcode:AHa12-000675,AHa12-000674; **Taxon:** scientificName: Microphorella praecox; order: Diptera; family: Dolichopodidae; scientificNameAuthorship: (Loew, 1864); **Location:** country: Finland; stateProvince: Ks; municipality: Kuusamo; locality: Jäkälämutka; verbatimCoordinates: 735797:361788; verbatimCoordinateSystem: ykj; decimalLatitude: 66.292; decimalLongitude: 29.623; geodeticDatum: WGS84; **Identification:** identifiedBy: Antti Haarto; **Event:** samplingProtocol: Malaise trap; eventDate: 2010-07-10/15; **Record Level:** collectionCode: Priv. Coll. A. Haarto; basisOfRecord: PreservedSpecimen

#### Distribution

Scattered records across north and central Europe from Finland to Italy (Pollet 2013), although the discovery of the closely related *Microphorella similis* Brooks & Ulrich, 2012 casts some doubt on old distribution data for *Microphorella praecox* (Brooks and Ulrich 2012). The presence of *Microphorella praecox* in Finland was considered doubtful by Kahanpää and Winqvist (2005). This new record confirms the presence of this species in Finland.

### Hydrophorus callosoma

Frey, 1915

http://www.faunaeur.org/full_results.php?id=135810

Hydrophorus wahlbergi var.(?) callosoma Frey 1915: 65 stat. rev.
Hydrophorus albosignatus Ringdahl 1919: 16 syn. nov.

#### Materials

- **Occurrence:** recordedBy: Wolter Hellén; individualCount: 1; sex: female; **Taxon:** scientificName: Hydrophorus callosoma; originalNameUsage: Hydrophorus wahlbergi var.(?) callosoma Frey, 1915; namePublishedIn: Frey, R. (1915) Zur kenntnis der Dipterenfauna Finlands. III. Dolichopodidae. Acta Societatis pro Fauna et Flora Fennica 40, 1–80+3 pl. Available from: http://www.biodiversitylibrary.org/page/5571884.; order: Diptera; family: Dolichopodidae; scientificNameAuthorship: Frey, 1915; **Location:** country: Russia; stateProvince: Murmansk district; municipality: Kandalaksha; verbatimLocality: Kantalaks; decimalLatitude: 67.20; decimalLongitude: 32.40; geodeticDatum: WGS84; **Identification:** identifiedBy: Jere Kahanpää; **Event:** eventDate: 1913-06-28; **Record Level:** collectionID: http://id.luomus.fi/HR.110; institutionCode: MZH; collectionCode: Diptera Fennica; basisOfRecord: PreservedSpecimen
- **Occurrence:** recordedBy: John Sahlberg; individualCount: 1; sex: female; **Taxon:** scientificName: Hydrophorus altivagus; originalNameUsage: Hydrophorus wahlbergi var.(?) callosoma Frey, 1915; namePublishedIn: Frey, R. (1915) Zur kenntnis der Dipterenfauna Finlands. III. Dolichopodidae. Acta Societatis pro Fauna et Flora Fennica 40, 1–80+3 pl. Available from: http://www.biodiversitylibrary.org/page/5571884.; order: Diptera; family: Dolichopodidae; scientificNameAuthorship: Aldrich, 1911; **Location:** country: Russia; stateProvince: Republic of Karelia; municipality: Kem'; verbatimLocality: Kem; decimalLatitude: 64.96; decimalLongitude: 34.60; geodeticDatum: WGS84; **Identification:** identifiedBy: Jere Kahanpää; **Event:** eventDate: 1869-07-30; **Record Level:** collectionID: http://id.luomus.fi/HR.110; institutionCode: MZH; collectionCode: Diptera Fennica; basisOfRecord: PreservedSpecimen
- **Occurrence:** recordedBy: Richard Frey; individualCount: 1; sex: male; **Taxon:** scientificName: Hydrophorus callosoma; order: Diptera; family: Dolichopodidae; scientificNameAuthorship: Frey, 1915; **Location:** country: Finland; stateProvince: PPe; municipality: Hailuoto; locality: Kirkkolahti; decimalLatitude: 65.00; decimalLongitude: 24.71; geodeticDatum: WGS84; coordinatePrecision: 3000; **Identification:** identifiedBy: Jere Kahanpää; **Event:** eventDate: 1947-07-16; **Record Level:** collectionID: http://id.luomus.fi/HR.110; institutionCode: MZH; collectionCode: Diptera Fennica; basisOfRecord: PreservedSpecimen
- **Occurrence:** recordedBy: Richard Frey; individualCount: 35; sex: 15 males, 20 females; **Taxon:** scientificName: Hydrophorus callosoma; order: Diptera; family: Dolichopodidae; scientificNameAuthorship: Frey, 1915; **Location:** country: Finland; stateProvince: PPe; municipality: Hailuoto; locality: Kirkkolahti; decimalLatitude: 65.00; decimalLongitude: 24.71; geodeticDatum: WGS84; coordinatePrecision: 3000; **Identification:** identifiedBy: Jere Kahanpää; **Event:** eventDate: 1947-07-12; **Record Level:** collectionID: http://id.luomus.fi/HR.110; institutionCode: MZH; collectionCode: Diptera Fennica; basisOfRecord: PreservedSpecimen
- **Occurrence:** recordedBy: Richard Frey; individualCount: 7; sex: 5 males, 2 females; **Taxon:** scientificName: Hydrophorus callosoma; order: Diptera; family: Dolichopodidae; scientificNameAuthorship: Frey, 1915; **Location:** country: Finland; stateProvince: PPe; municipality: Hailuoto; locality: Kirkkolahti; decimalLatitude: 65.00; decimalLongitude: 24.71; geodeticDatum: WGS84; coordinatePrecision: 3000; **Identification:** identifiedBy: Jere Kahanpää; **Event:** eventDate: 1947-07-11; **Record Level:** collectionID: http://id.luomus.fi/HR.110; institutionCode: MZH; collectionCode: Diptera Fennica; basisOfRecord: PreservedSpecimen
- **Occurrence:** recordedBy: Ragnar Storå; individualCount: 2; sex: 1 male, 1 female; **Taxon:** scientificName: Hydrophorus callosoma; order: Diptera; family: Dolichopodidae; scientificNameAuthorship: Frey, 1915; **Location:** country: Finland; stateProvince: KP; municipality: Pietarsaari; decimalLatitude: 63.690; decimalLongitude: 22.670; geodeticDatum: WGS84; coordinatePrecision: 1000; **Identification:** identifiedBy: Jere Kahanpää; **Event:** eventDate: 1953-01-01/05-01; **Record Level:** collectionID: http://id.luomus.fi/HR.110; institutionCode: MZH; collectionCode: Diptera Fennica; basisOfRecord: PreservedSpecimen
- **Occurrence:** recordedBy: Ragnar Storå; individualCount: 1; sex: female; **Taxon:** scientificName: Hydrophorus callosoma; order: Diptera; family: Dolichopodidae; scientificNameAuthorship: Frey, 1915; **Location:** country: Finland; stateProvince: KP; municipality: Pedersöre; decimalLatitude: 63.650; decimalLongitude: 22.810; geodeticDatum: WGS84; coordinatePrecision: 20000; **Identification:** identifiedBy: Jere Kahanpää; **Event:** eventDate: 1953-05-01; **Record Level:** collectionID: http://id.luomus.fi/HR.110; institutionCode: MZH; collectionCode: Diptera Fennica; basisOfRecord: PreservedSpecimen
- **Occurrence:** recordedBy: Ragnar Storå; individualCount: 1; sex: female; **Taxon:** scientificName: Hydrophorus callosoma; order: Diptera; family: Dolichopodidae; scientificNameAuthorship: Frey, 1915; **Location:** country: Finland; stateProvince: KP; municipality: Pedersöre; decimalLatitude: 63.650; decimalLongitude: 22.810; geodeticDatum: WGS84; coordinatePrecision: 20000; **Identification:** identifiedBy: Jere Kahanpää; **Event:** eventDate: 1953-08-14; **Record Level:** collectionID: http://id.luomus.fi/HR.110; institutionCode: MZH; collectionCode: Diptera Fennica; basisOfRecord: PreservedSpecimen
- **Occurrence:** recordedBy: Ragnar Storå; individualCount: 1; sex: male; **Taxon:** scientificName: Hydrophorus callosoma; order: Diptera; family: Dolichopodidae; scientificNameAuthorship: Frey, 1915; **Location:** country: Finland; stateProvince: KP; municipality: Pietarsaari; decimalLatitude: 63.690; decimalLongitude: 22.670; geodeticDatum: WGS84; coordinatePrecision: 1000; **Identification:** identifiedBy: Jere Kahanpää; **Event:** eventDate: 1953-08-15; **Record Level:** collectionID: http://id.luomus.fi/HR.110; institutionCode: MZH; collectionCode: Diptera Fennica; basisOfRecord: PreservedSpecimen
- **Occurrence:** recordedBy: Ragnar Storå; individualCount: 2; sex: males; **Taxon:** scientificName: Hydrophorus callosoma; order: Diptera; family: Dolichopodidae; scientificNameAuthorship: Frey, 1915; **Location:** country: Finland; stateProvince: KP; municipality: Pietarsaari; decimalLatitude: 63.690; decimalLongitude: 22.670; geodeticDatum: WGS84; coordinatePrecision: 1000; **Identification:** identifiedBy: Jere Kahanpää; **Event:** eventDate: 1953-08-16; **Record Level:** collectionID: http://id.luomus.fi/HR.110; institutionCode: MZH; collectionCode: Diptera Fennica; basisOfRecord: PreservedSpecimen
- **Occurrence:** recordedBy: Ragnar Storå; individualCount: 9; sex: 2 males, 7 females; **Taxon:** scientificName: Hydrophorus callosoma; order: Diptera; family: Dolichopodidae; scientificNameAuthorship: Frey, 1915; **Location:** country: Finland; stateProvince: KP; municipality: Pietarsaari; decimalLatitude: 63.690; decimalLongitude: 22.670; geodeticDatum: WGS84; coordinatePrecision: 1000; **Identification:** identifiedBy: Jere Kahanpää; **Event:** eventDate: 1953-08-19; **Record Level:** collectionID: http://id.luomus.fi/HR.110; institutionCode: MZH; collectionCode: Diptera Fennica; basisOfRecord: PreservedSpecimen
- **Occurrence:** recordedBy: Ragnar Storå; individualCount: 7; sex: 3 males, 4 females; **Taxon:** scientificName: Hydrophorus callosoma; order: Diptera; family: Dolichopodidae; scientificNameAuthorship: Frey, 1915; **Location:** country: Finland; stateProvince: KP; municipality: Pietarsaari; decimalLatitude: 63.690; decimalLongitude: 22.670; geodeticDatum: WGS84; coordinatePrecision: 1000; **Identification:** identifiedBy: Jere Kahanpää; **Event:** eventDate: 1953-08-20; **Record Level:** collectionID: http://id.luomus.fi/HR.110; institutionCode: MZH; collectionCode: Diptera Fennica; basisOfRecord: PreservedSpecimen
- **Occurrence:** recordedBy: Ragnar Storå; individualCount: 1; sex: female; **Taxon:** scientificName: Hydrophorus callosoma; order: Diptera; family: Dolichopodidae; scientificNameAuthorship: Frey, 1915; **Location:** country: Finland; stateProvince: KP; municipality: Pietarsaari; decimalLatitude: 63.690; decimalLongitude: 22.670; geodeticDatum: WGS84; coordinatePrecision: 1000; **Identification:** identifiedBy: Jere Kahanpää; **Event:** eventDate: 1953-08-22; **Record Level:** collectionID: http://id.luomus.fi/HR.110; institutionCode: MZH; collectionCode: Diptera Fennica; basisOfRecord: PreservedSpecimen
- **Occurrence:** recordedBy: Ragnar Storå; individualCount: 2; sex: 1 male, 1 female; **Taxon:** scientificName: Hydrophorus callosoma; order: Diptera; family: Dolichopodidae; scientificNameAuthorship: Frey, 1915; **Location:** country: Finland; stateProvince: KP; municipality: Pietarsaari; decimalLatitude: 63.690; decimalLongitude: 22.670; geodeticDatum: WGS84; coordinatePrecision: 1000; **Identification:** identifiedBy: Jere Kahanpää; **Event:** eventDate: 1953-08-29; **Record Level:** collectionID: http://id.luomus.fi/HR.110; institutionCode: MZH; collectionCode: Diptera Fennica; basisOfRecord: PreservedSpecimen
- **Occurrence:** recordedBy: Ragnar Storå; individualCount: 4; sex: 2 males, 2 females; **Taxon:** scientificName: Hydrophorus callosoma; order: Diptera; family: Dolichopodidae; scientificNameAuthorship: Frey, 1915; **Location:** country: Finland; stateProvince: KP; municipality: Pietarsaari; decimalLatitude: 63.690; decimalLongitude: 22.670; geodeticDatum: WGS84; coordinatePrecision: 1000; **Identification:** identifiedBy: Jere Kahanpää; **Event:** eventDate: 1953-09-05; **Record Level:** collectionID: http://id.luomus.fi/HR.110; institutionCode: MZH; collectionCode: Diptera Fennica; basisOfRecord: PreservedSpecimen
- **Occurrence:** recordedBy: Ragnar Storå; individualCount: 1; sex: female; **Taxon:** scientificName: Hydrophorus callosoma; order: Diptera; family: Dolichopodidae; scientificNameAuthorship: Frey, 1915; **Location:** country: Finland; stateProvince: KP; municipality: Pietarsaari; decimalLatitude: 63.690; decimalLongitude: 22.670; geodeticDatum: WGS84; coordinatePrecision: 1000; **Identification:** identifiedBy: Jere Kahanpää; **Event:** eventDate: 1953-09-20; **Record Level:** collectionID: http://id.luomus.fi/HR.110; institutionCode: MZH; collectionCode: Diptera Fennica; basisOfRecord: PreservedSpecimen
- **Occurrence:** recordedBy: Jere Kahanpää; individualCount: 1; sex: male; otherCatalogNumbers: labelcode: jka-05-00377; **Taxon:** scientificName: Hydrophorus callosoma; order: Diptera; family: Dolichopodidae; scientificNameAuthorship: Frey, 1915; **Location:** country: Finland; stateProvince: PPe; municipality: Hailuoto; locality: Santosenmatala; verbatimCoordinates: 72135:34085; verbatimCoordinateSystem: ykj; decimalLatitude: 65.006; decimalLongitude: 25.057; geodeticDatum: WGS84; coordinatePrecision: 200; **Identification:** identifiedBy: Jere Kahanpää; **Event:** samplingProtocol: sweep netting; eventDate: 2005-06-04; **Record Level:** collectionCode: Priv. Coll. J. Kahanpää; basisOfRecord: PreservedSpecimen
- **Occurrence:** recordedBy: Jere Kahanpää; individualCount: 17; sex: 8 males, 9 females; otherCatalogNumbers: labelcode: jka-05-04568, 04566, 04595, 04596, 04573, 04570, -04575, -04583, -04588, -04578, -04569, jka-05-04581, jka-05-04582, jka-05-04584, jka-05-04577, jka-05-04567, jka-05-04574; **Taxon:** scientificName: Hydrophorus callosoma; order: Diptera; family: Dolichopodidae; scientificNameAuthorship: Frey, 1915; **Location:** country: Finland; stateProvince: PPe; municipality: Lumijoki; locality: Varjakka, Karinpää; verbatimCoordinates: 72031:34090; verbatimCoordinateSystem: ykj; decimalLatitude: 64.913; decimalLongitude: 25.075; geodeticDatum: WGS84; coordinatePrecision: 200; **Identification:** identifiedBy: Jere Kahanpää; **Event:** samplingProtocol: sweep netting; eventDate: 2005-09-01; **Record Level:** collectionCode: Priv. Coll. J. Kahanpää; basisOfRecord: PreservedSpecimen
- **Occurrence:** recordedBy: Jere Kahanpää; individualCount: 1; sex: male; otherCatalogNumbers: labelcode: jka06-02709; **Taxon:** scientificName: Hydrophorus callosoma; order: Diptera; family: Dolichopodidae; scientificNameAuthorship: Frey, 1915; **Location:** country: Finland; stateProvince: PPe; municipality: Lumijoki; locality: Varjakka, Karinpää; verbatimCoordinates: 72031:34090; verbatimCoordinateSystem: ykj; decimalLatitude: 64.913; decimalLongitude: 25.075; geodeticDatum: WGS84; coordinatePrecision: 200; **Identification:** identifiedBy: Jere Kahanpää; **Event:** samplingProtocol: sweep netting; eventDate: 2006-07-09; **Record Level:** collectionCode: Priv. Coll. J. Kahanpää; basisOfRecord: PreservedSpecimen
- **Occurrence:** recordedBy: Jere Kahanpää; individualCount: 1; sex: male; **Taxon:** scientificName: Hydrophorus callosoma; order: Diptera; family: Dolichopodidae; scientificNameAuthorship: Frey, 1915; **Location:** country: Finland; stateProvince: KP; municipality: Kalajoki; locality: Äijänsäikkä M2; verbatimCoordinates: 713499:334989; verbatimCoordinateSystem: ykj; decimalLatitude: 64.281; decimalLongitude: 23.897; geodeticDatum: WGS84; coordinatePrecision: 100; **Identification:** identifiedBy: Jere Kahanpää; **Event:** samplingProtocol: Malaise trap; eventDate: 2005-06-03/07-03; **Record Level:** collectionCode: Priv. Coll. J. Kahanpää; basisOfRecord: PreservedSpecimen
- **Occurrence:** recordedBy: Jere Kahanpää; individualCount: 1; sex: male; otherCatalogNumbers: labelcode: jka-05-02637; **Taxon:** scientificName: Hydrophorus callosoma; order: Diptera; family: Dolichopodidae; scientificNameAuthorship: Frey, 1915; **Location:** country: Finland; stateProvince: KP; municipality: Kalajoki; locality: Äijänsäikkä; verbatimCoordinates: 71355:33499; verbatimCoordinateSystem: ykj; decimalLatitude: 64.286; decimalLongitude: 23.897; geodeticDatum: WGS84; coordinatePrecision: 200; **Identification:** identifiedBy: Jere Kahanpää; **Event:** samplingProtocol: sweep netting; eventDate: 2005-07-30; **Record Level:** collectionCode: Priv. Coll. J. Kahanpää; basisOfRecord: PreservedSpecimen
- **Occurrence:** recordedBy: Jere Kahanpää; individualCount: 1; sex: female; otherCatalogNumbers: labelcode: jka-05-04561; **Taxon:** scientificName: Hydrophorus callosoma; order: Diptera; family: Dolichopodidae; scientificNameAuthorship: Frey, 1915; **Location:** country: Finland; stateProvince: KP; municipality: Kalajoki; locality: Kalajokisuu; verbatimCoordinates: 71350:33499; verbatimCoordinateSystem: ykj; decimalLatitude: 64.282; decimalLongitude: 23.898; geodeticDatum: WGS84; coordinatePrecision: 200; **Identification:** identifiedBy: Jere Kahanpää; **Event:** samplingProtocol: sweep netting; eventDate: 2005-09-01; **Record Level:** collectionCode: Priv. Coll. J. Kahanpää; basisOfRecord: PreservedSpecimen

#### Diagnosis

*Hydrophorus callosoma* is externally most like *Hydrophorus altivagus* Aldrich, 1911 (=*Hydrophorus wahlgreni* Frey, 1915). Both sexes can be identified by the armature on fore femora: there is only a single short row of 6-13 small ventral spines in the basal third of the fore femur. Anteroventral spines missing or at most 1-3 very short ones present (usually completely absent in females). *Hydrophorus altivagus* has a row of strong anteroventral spines reaching the apical third of the femur in addition to the ventral spines. Other differences between the two species are tabulated in Table 1.

#### Distribution

Sweden, Finland and North-Western Russia (Murmansk and Archangelsk Oblasts).

#### Discussion

Richard Frey described *Hydrophorus callosoma* as a variety of on the basis of two female specimens. He did, however, express doubt about it's true status. The name has later been synonymed with *Hydrophorus wahlberg* Frey, 1915 (Negrobov 1977), which in turn was synonymised with *Hydrophorus altivagus* Aldrich, 1911 (Hurley 1985).

The type material of *Hydrophorus callosoma* Frey consists of two females deposited in MZH. The lectotype (here designated), a female from Kantalaks (=Kandalaksha, Russia), is identical with *Hydrophorus albosignatus* Ringdahl, 1919 (Figs 1c, 2c, 3c). Frey's second type specimen from Kem belongs to *Hydrophorus altivagus*.

Frey's *Hydrophorus callosoma* type material was compared with a single female paralectotype of *Hydrophorus albosignatus* Ringdahl, 1919 (see Figs 1e, 2e, 3e). It does match the Kantalaks specimen and the description of the male provided by Ringdahl, plus all available figures of the two species, clearly indicate that *Hydrophorus albosignatus* Ringdahl is identical with *Hydrophorus callosoma* of Frey.

The Kantalaks specimen is here designated as the lectotype of *Hydrophorus callosoma* Frey, 1915. This choice is made because only this specimen does actually match Frey's original description "...near the previous species [=*Hydrophorus wahlbergi*], from which is differs by its much more brilliant coloration and the pale hairs on the sides of the abdomen" (Frey 1915). The Kem specimen—like all specimens of both species—have pale hairs on the ventral margin of the tergites only. Because of the distortion of the abdomen of this dry-pinned female, the ventral hairs are visible when viewed from above.

*Hydrophorus callosoma* Frey, 1915 is thus a senior synonym of *Hydrophorus albosignatus* Ringdahl, 1919 and becomes the valid name for this species (syn. nov.).

### Medetera belgica

Parent, 1936

http://www.faunaeur.org/full_results.php?id=135916

#### Materials

- **Occurrence:** recordedBy: E. J. Bonsdorff; individualCount: 1; sex: female; otherCatalogNumbers: labelcode: jka06-03068; **Taxon:** scientificName: Medetera belgica; order: Diptera; family: Dolichopodidae; scientificNameAuthorship: Parent, 1936; **Location:** country: Finland; stateProvince: V; municipality: Turku; decimalLatitude: 60.5; decimalLongitude: 22.3; geodeticDatum: WGS84; **Identification:** identifiedBy: Jere Kahanpää; **Record Level:** collectionID: http://id.luomus.fi/HR.110; institutionCode: MZH; collectionCode: Diptera Fennica; basisOfRecord: PreservedSpecimen
- **Occurrence:** recordedBy: Richard Frey; individualCount: 2; sex: 1 male, 1 female; otherCatalogNumbers: labelcode:jka06-03070, jka06-03067; **Taxon:** scientificName: Medetera belgica; order: Diptera; family: Dolichopodidae; scientificNameAuthorship: Parent, 1936; **Location:** country: Finland; stateProvince: V; municipality: Lohja; decimalLatitude: 60.2; decimalLongitude: 24.1; geodeticDatum: WGS84; **Identification:** identifiedBy: Jere Kahanpää; **Record Level:** collectionID: http://id.luomus.fi/HR.110; institutionCode: MZH; collectionCode: Diptera Fennica; basisOfRecord: PreservedSpecimen
- **Occurrence:** recordedBy: Anders Albrecht; individualCount: 1; sex: female; otherCatalogNumbers: labelcode:jka06-03071; **Taxon:** scientificName: Medetera belgica; order: Diptera; family: Dolichopodidae; scientificNameAuthorship: Parent, 1936; **Location:** country: Finland; stateProvince: V; municipality: Kiikala; decimalLatitude: 60.5; decimalLongitude: 23.6; geodeticDatum: WGS84; **Identification:** identifiedBy: Jere Kahanpää; **Event:** eventDate: 1980-06-22; **Record Level:** collectionID: http://id.luomus.fi/HR.110; institutionCode: MZH; collectionCode: Diptera Fennica; basisOfRecord: PreservedSpecimen
- **Occurrence:** recordedBy: Olli Autio; Jukka Salmela; individualCount: 1; sex: male; **Taxon:** scientificName: Medetera belgica; order: Diptera; family: Dolichopodidae; scientificNameAuthorship: Parent, 1936; **Location:** country: Finland; stateProvince: A; municipality: Jomala; locality: Björsby; verbatimCoordinates: 669627:311108; verbatimCoordinateSystem: ykj; decimalLatitude: 60.192; decimalLongitude: 19.978; geodeticDatum: WGS84; coordinatePrecision: 200; **Identification:** identifiedBy: Jere Kahanpää; **Event:** samplingProtocol: Malaise trap; eventDate: 2007-04-22/06-17; **Record Level:** collectionCode: Priv. Coll. J. Kahanpää; basisOfRecord: PreservedSpecimen
- **Occurrence:** recordedBy: J. Kahanpää; individualCount: 1; sex: male; otherCatalogNumbers: labelcode: jka07-03109; **Taxon:** scientificName: Medetera belgica; order: Diptera; family: Dolichopodidae; scientificNameAuthorship: Parent, 1936; **Location:** country: Finland; stateProvince: A; municipality: Saltvik; locality: Höckböle; verbatimCoordinates: 67164:31097; verbatimCoordinateSystem: ykj; decimalLatitude: 60.370; decimalLongitude: 19.915; geodeticDatum: WGS84; coordinatePrecision: 200; **Identification:** identifiedBy: Jere Kahanpää; **Event:** samplingProtocol: sweep netting; eventDate: 2007-06-13; **Record Level:** collectionCode: Priv. Coll. J. Kahanpää; basisOfRecord: PreservedSpecimen
- **Occurrence:** recordedBy: Olli Autio; Jukka Salmela; individualCount: 1; sex: male; **Taxon:** scientificName: Medetera belgica; order: Diptera; family: Dolichopodidae; scientificNameAuthorship: Parent, 1936; **Location:** country: Finland; stateProvince: A; municipality: Sund; locality: Bredmossen; verbatimCoordinates: 670452:312546; verbatimCoordinateSystem: ykj; decimalLatitude: 60.279; decimalLongitude: 20.220; geodeticDatum: WGS84; coordinatePrecision: 200; **Identification:** identifiedBy: Jere Kahanpää; **Event:** samplingProtocol: Malaise trap; eventDate: 2007-06-16/07-28; **Record Level:** collectionCode: Priv. Coll. J. Kahanpää; basisOfRecord: PreservedSpecimen
- **Occurrence:** recordedBy: Olli Autio; Jukka Salmela; individualCount: 3; sex: 1 male, 2 females; **Taxon:** scientificName: Medetera belgica; order: Diptera; family: Dolichopodidae; scientificNameAuthorship: Parent, 1936; **Location:** country: Finland; stateProvince: A; municipality: Saltvik; locality: Långbergsöda; verbatimCoordinates: 670761:312132; verbatimCoordinateSystem: ykj; decimalLatitude: 60.302; decimalLongitude: 20.140; geodeticDatum: WGS84; coordinatePrecision: 200; **Identification:** identifiedBy: Jere Kahanpää; **Event:** samplingProtocol: Malaise trap; eventDate: 2007-06-16/07-28; **Record Level:** collectionCode: Priv. Coll. J. Kahanpää; basisOfRecord: PreservedSpecimen
- **Occurrence:** recordedBy: Richard Frey; individualCount: 1; sex: male; otherCatalogNumbers: labelcode:jka06-03074; **Taxon:** scientificName: Medetera belgica; order: Diptera; family: Dolichopodidae; scientificNameAuthorship: Parent, 1936; **Location:** country: Finland; stateProvince: U; municipality: Hanko; locality: Tvärminne, Spikarna; decimalLatitude: 59.811; decimalLongitude: 23.205; geodeticDatum: WGS84; coordinatePrecision: 300; **Identification:** identifiedBy: Jere Kahanpää; **Event:** eventDate: 1920-07-28; **Record Level:** collectionID: http://id.luomus.fi/HR.110; institutionCode: MZH; collectionCode: Diptera Fennica; basisOfRecord: PreservedSpecimen
- **Occurrence:** recordedBy: Jari Flinck; individualCount: 1; sex: male; otherCatalogNumbers: labelcode:JF10-2527; **Taxon:** scientificName: Medetera belgica; order: Diptera; family: Dolichopodidae; scientificNameAuthorship: Parent, 1936; **Location:** country: Finland; stateProvince: U; municipality: Loviisa; locality: Rauhala; verbatimCoordinates: 6701979:8456973; verbatimCoordinateSystem: etrs-tm35fin; decimalLatitude: 60.452; decimalLongitude: 26.218; geodeticDatum: WGS84; coordinatePrecision: 200; **Identification:** identifiedBy: Marc Pollet; **Event:** samplingProtocol: Malaise trap; eventDate: 2010-06-26; **Record Level:** collectionCode: Priv. Coll.J. Flinck; basisOfRecord: PreservedSpecimen
- **Occurrence:** recordedBy: Ragnar Storå; individualCount: 1; sex: male; otherCatalogNumbers: labelcode:jka06-03072; **Taxon:** scientificName: Medetera belgica; order: Diptera; family: Dolichopodidae; scientificNameAuthorship: Parent, 1936; **Location:** country: Finland; stateProvince: PK; municipality: Pietarsaari; verbatimCoordinateSystem: ykj; decimalLatitude: 63.7; decimalLongitude: 22.7; geodeticDatum: WGS84; **Identification:** identifiedBy: Jere Kahanpää; **Event:** eventDate: 1957-07-20; **Record Level:** collectionID: http://id.luomus.fi/HR.110; institutionCode: MZH; collectionCode: Diptera Fennica; basisOfRecord: PreservedSpecimen
- **Occurrence:** recordedBy: Erik Thuneberg; individualCount: 1; sex: female; otherCatalogNumbers: labelcode:jka06-03069; **Taxon:** scientificName: Medetera belgica; order: Diptera; family: Dolichopodidae; scientificNameAuthorship: Parent, 1936; **Location:** country: Finland; stateProvince: ES; municipality: Joutseno; decimalLatitude: 61.1; decimalLongitude: 28.5; geodeticDatum: WGS84; **Identification:** identifiedBy: Jere Kahanpää; **Event:** eventDate: 1948-06-24; **Record Level:** collectionID: http://id.luomus.fi/HR.110; institutionCode: MZH; collectionCode: Diptera Fennica; basisOfRecord: PreservedSpecimen
- **Occurrence:** recordedBy: Lauri Tiensuu; individualCount: 1; sex: male; otherCatalogNumbers: labelcode:jka06-03073; **Taxon:** scientificName: Medetera belgica; order: Diptera; family: Dolichopodidae; scientificNameAuthorship: Parent, 1936; **Location:** country: Finland; stateProvince: EH; municipality: Lammi; decimalLatitude: 61.1; decimalLongitude: 25.0; geodeticDatum: WGS84; **Identification:** identifiedBy: Jere Kahanpää; **Event:** eventDate: 1955-07-31; **Record Level:** collectionID: http://id.luomus.fi/HR.110; institutionCode: MZH; collectionCode: Diptera Fennica; basisOfRecord: PreservedSpecimen

#### Distribution

New to Finland. Global distribution poorly known due to confusion with *Medetera muralis* (Fallén, 1823) (Pollet 2013).

#### Discussion

*Medetera belgica* was based on a single female Parent 1936. Negrobov and Stackelberg 1972 redescribed the species based on male specimens from the Kola Peninsula in Russia, some 2,500 km away, without studying the holotype of *Medetera belgica*. It is certainly possible that the Finnish specimens identified as *Medetera belgica* sensu Negrobov are not conspecific with Parent's original types. *Medetera belgica* was synonymised with *Medetera muralis* by Grichanov (2002), but this synonymy has been disputed (Pollet 2013). In Finnish material the leg colour characters and differences in male genitalia between the two species (*Medetera muralis* and *Medetera belgica* sensu Negrobov) are well defined and constant.

### Medetera fumida

Negrobov, 1967

http://www.faunaeur.org/full_results.php?id=135944

#### Materials

- **Occurrence:** recordedBy: Jere Kahanpää; individualCount: 2; sex: males; otherCatalogNumbers: labelcode:jka11-00182; **Taxon:** scientificName: Medetera fumida; order: Diptera; family: Dolichopodidae; scientificNameAuthorship: Negrobov, 1967; **Location:** country: Finland; stateProvince: U; municipality: Pukkila; locality: Venunmetsä; verbatimCoordinates: 67284:34159; verbatimCoordinateSystem: ykj; decimalLatitude: 60.658; decimalLongitude: 25.460; geodeticDatum: WGS84; coordinatePrecision: 1000; **Identification:** identifiedBy: Jere Kahanpää; **Event:** samplingProtocol: sweep netting; eventDate: 2011-06-11; **Record Level:** collectionCode: Priv. Coll. J. Kahanpää; basisOfRecord: PreservedSpecimen

#### Distribution

New to Finland. Previously known from Estonia and Russia (Negrobov 1991, Grichanov 2002b).

### Medetera prjachinae

Negrobov & Stackelberg, 1974

http://www.faunaeur.org/full_results.php?id=136007

#### Materials

- **Occurrence:** recordedBy: Jere Kahanpää; individualCount: 1; sex: male; **Taxon:** scientificName: Medetera prjachinae; order: Diptera; family: Dolichopodidae; scientificNameAuthorship: Negrobov & Stackelberg, 1974; **Location:** country: Finland; stateProvince: U; municipality: Pukkila; locality: Venunmetsä; verbatimCoordinates: 67284:34159; verbatimCoordinateSystem: ykj; decimalLatitude: 60.658; decimalLongitude: 25.460; geodeticDatum: WGS84; coordinatePrecision: 1000; **Identification:** identifiedBy: Jere Kahanpää; **Event:** samplingProtocol: sweep netting; eventDate: 2011-06-11; **Record Level:** collectionCode: Priv. Coll. J. Kahanpää; basisOfRecord: PreservedSpecimen

#### Distribution

New to Finland. Previously known from Russia (Archangelsk Oblast) and Estonia (Negrobov 1991, Grichanov 2002b).

### Medetera seguyi

Parent, 1926

http://www.faunaeur.org/full_results.php?id=136015

#### Materials

- **Occurrence:** recordedBy: Jevgeni Jakovlev; Jouni Penttinen; individualCount: 7; sex: 5 males, 1 female; **Taxon:** scientificName: Medetera seguyi; order: Diptera; family: Dolichopodidae; scientificNameAuthorship: Parent, 1926; **Location:** country: Finland; stateProvince: EH; municipality: Lammi; locality: Kotisten aarnialue; verbatimCoordinates: 6794055:3396410; verbatimCoordinateSystem: ykj; decimalLatitude: 61.241; decimalLongitude: 25.067; geodeticDatum: WGS84; **Identification:** identifiedBy: Jere Kahanpää; **Event:** samplingProtocol: emergence trap; eventDate: 2006-05-22/06-27; **Record Level:** collectionCode: Priv. Coll. J. Kahanpää; basisOfRecord: PreservedSpecimen
- **Occurrence:** recordedBy: Jevgeni Jakovlev; Jouni Penttinen; individualCount: 1; sex: male; **Taxon:** scientificName: Medetera seguyi; order: Diptera; family: Dolichopodidae; scientificNameAuthorship: Parent, 1926; **Location:** country: Finland; stateProvince: EH; municipality: Lammi; locality: Kotisten aarnialue A4; verbatimCoordinates: 6794369:3396424; verbatimCoordinateSystem: ykj; decimalLatitude: 61.244; decimalLongitude: 25.068; geodeticDatum: WGS84; **Identification:** identifiedBy: Jere Kahanpää; **Event:** samplingProtocol: emergence trap; eventDate: 2006-06-28/08-02; **Record Level:** collectionCode: Priv. Coll. J. Kahanpää; basisOfRecord: PreservedSpecimen
- **Occurrence:** recordedBy: Jevgeni Jakovlev; Jouni Penttinen; individualCount: 11; sex: 6 males, 5 females; **Taxon:** scientificName: Medetera seguyi; order: Diptera; family: Dolichopodidae; scientificNameAuthorship: Parent, 1926; **Location:** country: Finland; stateProvince: EH; municipality: Lammi; locality: Kotinen AB-2-I; decimalLatitude: 61.24; decimalLongitude: 25.06; geodeticDatum: WGS84; **Identification:** identifiedBy: Jere Kahanpää; **Event:** samplingProtocol: emergence trap; eventDate: 2006-05-24/06-27; **Record Level:** collectionCode: Priv. Coll. J. Kahanpää; basisOfRecord: PreservedSpecimen
- **Occurrence:** recordedBy: Jevgeni Jakovlev; Jouni Penttinen; individualCount: 2; sex: 2 males; **Taxon:** scientificName: Medetera seguyi; order: Diptera; family: Dolichopodidae; scientificNameAuthorship: Parent, 1926; **Location:** country: Finland; stateProvince: U; municipality: Lapinjärvi; locality: 2-II,III,IV,V; decimalLatitude: 60.6; decimalLongitude: 26.2; geodeticDatum: WGS84; **Identification:** identifiedBy: Jere Kahanpää; **Event:** samplingProtocol: emergence trap; eventDate: 2005-06-30/09-27; **Record Level:** collectionCode: Priv. Coll. J. Kahanpää; basisOfRecord: PreservedSpecimen
- **Occurrence:** recordedBy: Jevgeni Jakovlev; Jouni Penttinen; individualCount: 3; sex: 3 males; **Taxon:** scientificName: Medetera seguyi; order: Diptera; family: Dolichopodidae; scientificNameAuthorship: Parent, 1926; **Location:** country: Finland; stateProvince: U; municipality: Lapinjärvi; locality: 2-II,III,IV,V; decimalLatitude: 60.6; decimalLongitude: 26.2; geodeticDatum: WGS84; **Identification:** identifiedBy: Jere Kahanpää; **Event:** samplingProtocol: emergence trap; eventDate: 2005-04-25/06-30; **Record Level:** collectionCode: Priv. Coll. J. Kahanpää; basisOfRecord: PreservedSpecimen
- **Occurrence:** recordedBy: Jevgeni Jakovlev; Jouni Penttinen; individualCount: 2; sex: 1 male, 1 female; **Taxon:** scientificName: Medetera seguyi; order: Diptera; family: Dolichopodidae; scientificNameAuthorship: Parent, 1926; **Location:** country: Finland; stateProvince: U; municipality: Lapinjärvi; locality: 3-I; decimalLatitude: 60.6; decimalLongitude: 26.2; geodeticDatum: WGS84; **Identification:** identifiedBy: Jere Kahanpää; **Event:** samplingProtocol: emergence trap; eventDate: 2005-04-25/06-30; **Record Level:** collectionCode: Priv. Coll. J. Kahanpää; basisOfRecord: PreservedSpecimen
- **Occurrence:** recordedBy: Jevgeni Jakovlev; Jouni Penttinen; individualCount: 2; sex: 2 males; **Taxon:** scientificName: Medetera seguyi; order: Diptera; family: Dolichopodidae; scientificNameAuthorship: Parent, 1926; **Location:** country: Finland; stateProvince: EH; municipality: Lammi; locality: Kotinen 12-II; decimalLatitude: 61.24; decimalLongitude: 25.06; geodeticDatum: WGS84; **Identification:** identifiedBy: Jere Kahanpää; **Event:** samplingProtocol: emergence trap; eventDate: 2005-06-10/30; **Record Level:** collectionCode: Priv. Coll. J. Kahanpää; basisOfRecord: PreservedSpecimen

#### Distribution

New to Finland. Previously reported from Norway, Belgium, Switzerland, France, south Russia (Negrobov 1991, Naglis 2009, Pollet 2013), but this taxon might involve a group of closely related species.

### Medetera zinovjevi

Negrobov, 1967

http://www.faunaeur.org/full_results.php?id=136039

#### Materials

- **Occurrence:** recordedBy: Jukka Salmela; individualCount: 1; sex: male; **Taxon:** scientificName: Medetera zinovjevi; order: Diptera; family: Dolichopodidae; scientificNameAuthorship: Negrobov, 1967; **Location:** country: Finland; stateProvince: Ks; municipality: Taivalkoski; locality: Syväoja; verbatimCoordinates: 7299627:3560581; verbatimCoordinateSystem: ykj; decimalLatitude: 65.785; decimalLongitude: 28.319; geodeticDatum: WGS84; coordinatePrecision: 50; **Identification:** identifiedBy: Jere Kahanpää; **Event:** samplingProtocol: Malaise trap; eventDate: 2006-05-31/07-03; **Record Level:** collectionCode: Priv. Coll. J. Kahanpää; basisOfRecord: PreservedSpecimen
- **Occurrence:** recordedBy: Jukka Salmela; individualCount: 1; sex: male; **Taxon:** scientificName: Medetera zinovjevi; order: Diptera; family: Dolichopodidae; scientificNameAuthorship: Negrobov, 1967; **Location:** country: Finland; stateProvince: Ks; municipality: Taivalkoski; locality: Kylmäoja; verbatimCoordinates: 7275293:3554865; verbatimCoordinateSystem: ykj; decimalLatitude: 65.568; decimalLongitude: 28.185; geodeticDatum: WGS84; coordinatePrecision: 50; **Identification:** identifiedBy: Jere Kahanpää; **Event:** samplingProtocol: Malaise trap; eventDate: 2006-05-31/07-03; **Record Level:** collectionCode: Priv. Coll. J. Kahanpää; basisOfRecord: PreservedSpecimen

#### Distribution

New to Finland. Previously known from Russia, Estonia, Lithuania and Norway (Negrobov 1991, Jonassen 1998, Grichanov 2002, Pakalniškis et al. 2006).

### Dolichopus annulitarsis

Ringdahl, 1920

http://www.faunaeur.org/full_results.php?id=135463

#### Materials

- **Occurrence:** recordedBy: Antti Haarto; individualCount: 1; sex: male; otherCatalogNumbers: labelcode:AHa12-000157; **Taxon:** scientificName: Dolichopus annulitarsis; order: Diptera; family: Dolichopodidae; scientificNameAuthorship: Ringdahl, 1920; **Location:** country: Finland; stateProvince: EnL; municipality: Enontekiö; locality: Kilpisjärvi, Urtasjoki; verbatimCoordinates: 769206:326363; verbatimCoordinateSystem: ykj; decimalLatitude: 69.206; decimalLongitude: 21.024; geodeticDatum: WGS84; **Identification:** identifiedBy: Jere Kahanpää; **Event:** eventDate: 2009-07-09/15; **Record Level:** collectionCode: Priv. Coll. A. Haarto; basisOfRecord: PreservedSpecimen

#### Distribution

New to Finland. A rare species previously known from Sweden and Alaska (Ringdahl 1920, Pollet 2013).

### Dolichopus cilifemoratus

Macquart, 1827

http://www.faunaeur.org/full_results.php?id=135484

= Dolichopus cilifemoratus Macquart, 1827
= Dolichopus pseudocilifemoratus Stackelberg, 1930

#### Materials

- **Occurrence:** recordedBy: Olli Autio; Jukka Salmela; individualCount: 1; sex: male; **Taxon:** scientificName: Dolichopus cilifemoratus; order: Diptera; family: Dolichopodidae; scientificNameAuthorship: Macquart, 1827; **Location:** country: Finland; stateProvince: A; municipality: Eckerö; locality: Holmträsket; verbatimCoordinates: 670517:309246; verbatimCoordinateSystem: ykj; decimalLatitude: 60.253; decimalLongitude: 19.627; geodeticDatum: WGS84; coordinatePrecision: 200; **Identification:** identifiedBy: Jere Kahanpää; **Event:** samplingProtocol: Malaise trap; eventDate: 2007-06-15/07-27; **Record Level:** collectionCode: Priv. Coll. J. Kahanpää; basisOfRecord: PreservedSpecimen

#### Distribution

New to Finland. Widespread in Europe, but many records should be confirmed due to the frequent misuse of this name for *Dolichopus trivialis* Haliday, 1832 (Pollet 2013).

#### Discussion

This name has a confused history of use, which can be traced back to von Stackelberg (1930), who erroneously synonymised Macquart's *Dolichopus cilifemoratus* with the species now called *Dolichopus trivialis* Haliday, 1832. He then created a new name, *Dolichopus pseudocilifemoratus* Stackelberg, 1930. Subsequent authors have often repeated Stackelberg's mistake. This record represents the first time the genuine *Dolichopus cilifemoratus* of Macquart is found in Finland.

### Dolichopus costalis

Frey, 1915

http://www.faunaeur.org/full_results.php?id=135495

#### Materials

- **Occurrence:** recordedBy: Jukka Salmela; Jari Ilmonen; individualCount: 3; sex: 2 males, 1 female; **Taxon:** scientificName: Dolichopus costalis; order: Diptera; family: Dolichopodidae; scientificNameAuthorship: Frey, 1915; **Location:** country: Finland; stateProvince: PPp; municipality: Tervola; locality: Karhakkamaanjänkä; verbatimCoordinates: 7346254:3415785; verbatimCoordinateSystem: ykj; decimalLatitude: 66.197; decimalLongitude: 25.127; geodeticDatum: WGS84; coordinatePrecision: 50; **Identification:** identifiedBy: Jere Kahanpää; **Event:** samplingProtocol: Malaise trap; eventDate: 2004-05-29/06/28; **Record Level:** collectionCode: Priv. Coll. J. Kahanpää; basisOfRecord: PreservedSpecimen
- **Occurrence:** recordedBy: Jukka Salmela; Jari Ilmonen; individualCount: 2; sex: 1 male, 1 female; **Taxon:** scientificName: Dolichopus costalis; order: Diptera; family: Dolichopodidae; scientificNameAuthorship: Frey, 1915; **Location:** country: Finland; stateProvince: PPp; municipality: Tervola; locality: Keskipalo; verbatimCoordinates: 7344806:3415122; verbatimCoordinateSystem: ykj; decimalLatitude: 66.184; decimalLongitude: 25.113; geodeticDatum: WGS84; coordinatePrecision: 50; **Identification:** identifiedBy: Jere Kahanpää; **Event:** samplingProtocol: Malaise trap; eventDate: 2004-05-29/06/28; **Record Level:** collectionCode: Priv. Coll. J. Kahanpää; basisOfRecord: PreservedSpecimen
- **Occurrence:** recordedBy: Jukka Salmela; Jari Ilmonen; individualCount: 2; sex: males; **Taxon:** scientificName: Dolichopus costalis; order: Diptera; family: Dolichopodidae; scientificNameAuthorship: Frey, 1915; **Location:** country: Finland; stateProvince: PPp; municipality: Tervola; locality: Hirviaapa; verbatimCoordinates: 7347499:3418464; verbatimCoordinateSystem: ykj; decimalLatitude: 66.209; decimalLongitude: 25.185; geodeticDatum: WGS84; coordinatePrecision: 50; **Identification:** identifiedBy: Jere Kahanpää; **Event:** samplingProtocol: Malaise trap; eventDate: 2004-05-29/06/28; **Record Level:** collectionCode: Priv. Coll. J. Kahanpää; basisOfRecord: PreservedSpecimen
- **Occurrence:** recordedBy: Jukka Salmela; Jari Ilmonen; individualCount: 1; sex: female; **Taxon:** scientificName: Dolichopus costalis; order: Diptera; family: Dolichopodidae; scientificNameAuthorship: Frey, 1915; **Location:** country: Finland; stateProvince: PPp; municipality: Tervola; locality: Mulkosilmälampi; verbatimCoordinates: 7348838:3408017; verbatimCoordinateSystem: ykj; decimalLatitude: 66.218; decimalLongitude: 24.953; geodeticDatum: WGS84; coordinatePrecision: 50; **Identification:** identifiedBy: Jere Kahanpää; **Event:** samplingProtocol: Malaise trap; eventDate: 2004-05-29/06/28; **Record Level:** collectionCode: Priv. Coll. J. Kahanpää; basisOfRecord: PreservedSpecimen
- **Occurrence:** recordedBy: Jukka Salmela; Jari Ilmonen; individualCount: 1; sex: male; **Taxon:** scientificName: Dolichopus costalis; order: Diptera; family: Dolichopodidae; scientificNameAuthorship: Frey, 1915; **Location:** country: Finland; stateProvince: PPp; municipality: Tervola; locality: Hirviaapa; verbatimCoordinates: 7347499:3418464; verbatimCoordinateSystem: ykj; decimalLatitude: 66.209; decimalLongitude: 25.185; geodeticDatum: WGS84; coordinatePrecision: 50; **Identification:** identifiedBy: Jere Kahanpää; **Event:** samplingProtocol: Malaise trap; eventDate: 2004-06-28/08-02; **Record Level:** collectionCode: Priv. Coll. J. Kahanpää; basisOfRecord: PreservedSpecimen
- **Occurrence:** recordedBy: Jukka Salmela; Jari Ilmonen; individualCount: 5; sex: 3 males, 2 females; **Taxon:** scientificName: Dolichopus costalis; order: Diptera; family: Dolichopodidae; scientificNameAuthorship: Frey, 1915; **Location:** country: Finland; stateProvince: PPp; municipality: Tervola; locality: Karhakkamaanjänkä; verbatimCoordinates: 7346254:3415785; verbatimCoordinateSystem: ykj; decimalLatitude: 66.197; decimalLongitude: 25.127; geodeticDatum: WGS84; coordinatePrecision: 50; **Identification:** identifiedBy: Jere Kahanpää; **Event:** samplingProtocol: Malaise trap; eventDate: 2004-06-28/08-02; **Record Level:** collectionCode: Priv. Coll. J. Kahanpää; basisOfRecord: PreservedSpecimen
- **Occurrence:** recordedBy: Jukka Salmela; Jari Ilmonen; individualCount: 1; sex: male; **Taxon:** scientificName: Dolichopus costalis; order: Diptera; family: Dolichopodidae; scientificNameAuthorship: Frey, 1915; **Location:** country: Finland; stateProvince: PPp; municipality: Tervola; locality: Keskipalo; verbatimCoordinates: 7344806:3415122; verbatimCoordinateSystem: ykj; decimalLatitude: 66.184; decimalLongitude: 25.113; geodeticDatum: WGS84; coordinatePrecision: 50; **Identification:** identifiedBy: Jere Kahanpää; **Event:** samplingProtocol: Malaise trap; eventDate: 2004-06-28/08-02; **Record Level:** collectionCode: Priv. Coll. J. Kahanpää; basisOfRecord: PreservedSpecimen
- **Occurrence:** recordedBy: Jukka Salmela; Jari Ilmonen; individualCount: 1; sex: female; **Taxon:** scientificName: Dolichopus costalis; order: Diptera; family: Dolichopodidae; scientificNameAuthorship: Frey, 1915; **Location:** country: Finland; stateProvince: PPp; municipality: Tervola; locality: Mulkosilmälampi; verbatimCoordinates: 7348838:3408017; verbatimCoordinateSystem: ykj; decimalLatitude: 66.218; decimalLongitude: 24.953; geodeticDatum: WGS84; coordinatePrecision: 50; **Identification:** identifiedBy: Jere Kahanpää; **Event:** samplingProtocol: Malaise trap; eventDate: 2004-06-28/08-02; **Record Level:** collectionCode: Priv. Coll. J. Kahanpää; basisOfRecord: PreservedSpecimen
- **Occurrence:** recordedBy: Jere Kahanpää; individualCount: 1; sex: male; **Taxon:** scientificName: Dolichopus costalis; order: Diptera; family: Dolichopodidae; scientificNameAuthorship: Frey, 1915; **Location:** country: Finland; stateProvince: PPp; municipality: Tervola; locality: Hirviaapa; verbatimCoordinates: 7347499:3418464; verbatimCoordinateSystem: ykj; decimalLatitude: 66.209; decimalLongitude: 25.185; geodeticDatum: WGS84; coordinatePrecision: 50; **Identification:** identifiedBy: Jere Kahanpää; **Event:** samplingProtocol: sweep netting; eventDate: 2006-07-08; **Record Level:** collectionCode: Priv. Coll. J. Kahanpää; basisOfRecord: PreservedSpecimen
- **Occurrence:** recordedBy: Jere Kahanpää; individualCount: 1; sex: male; **Taxon:** scientificName: Dolichopus costalis; order: Diptera; family: Dolichopodidae; scientificNameAuthorship: Frey, 1915; **Location:** country: Finland; stateProvince: PPp; municipality: Tervola; locality: Mulkosilmälampi; verbatimCoordinates: 734884:340802; verbatimCoordinateSystem: ykj; decimalLatitude: 66.219; decimalLongitude: 24.953; geodeticDatum: WGS84; coordinatePrecision: 100; **Identification:** identifiedBy: Jere Kahanpää; **Event:** samplingProtocol: sweep netting; eventDate: 2006-07-09; **Record Level:** collectionCode: Priv. Coll. J. Kahanpää; basisOfRecord: PreservedSpecimen

#### Biology

A species of eutrophic calcareous spring-fed fens in northern Finland.

#### Distribution

New to (present-day) Finland. Previously recorded as Finnish based on specimens from areas ceded to Russia after the second world war (Kahanpää and Grichanov 2004). Also reported from Sweden, northern Russia, Mongolia and (doubtfully) in the Oriental region (Frey 1915, Grichanov 2002, Pollet 2013).

### Dolichopus lancearius

Hedström, 1966

http://www.faunaeur.org/full_results.php?id=135529

#### Materials

- **Occurrence:** recordedBy: Jukka Salmela; Jari Ilmonen; individualCount: 3; sex: 2 males, 1 female; **Taxon:** scientificName: Dolichopus lancearius; order: Diptera; family: Dolichopodidae; scientificNameAuthorship: Hedström, 1966; **Location:** country: Finland; stateProvince: PPp; municipality: Tervola; locality: Ruuttulampi III; verbatimCoordinates: 7347734:3409699; verbatimCoordinateSystem: ykj; decimalLatitude: 66.209; decimalLongitude: 24.991; geodeticDatum: WGS84; coordinatePrecision: 100; **Identification:** identifiedBy: Jere Kahanpää; **Event:** samplingProtocol: Malaise trap; eventDate: 2004-06-28/08-02; **Record Level:** collectionCode: Priv. Coll. J. Kahanpää; basisOfRecord: PreservedSpecimen
- **Occurrence:** recordedBy: Jukka Salmela; Jari Ilmonen; individualCount: 3; sex: 3 females; **Taxon:** scientificName: Dolichopus lancearius; order: Diptera; family: Dolichopodidae; scientificNameAuthorship: Hedström, 1966; **Location:** country: Finland; stateProvince: PPp; municipality: Tervola; locality: Hirviaapa; verbatimCoordinates: 7347499:3418464; verbatimCoordinateSystem: ykj; decimalLatitude: 66.209; decimalLongitude: 25.185; geodeticDatum: WGS84; coordinatePrecision: 100; **Identification:** identifiedBy: Jere Kahanpää; **Event:** samplingProtocol: Malaise trap; eventDate: 2004-06-28/08-02; **Record Level:** collectionCode: Priv. Coll. J. Kahanpää; basisOfRecord: PreservedSpecimen
- **Occurrence:** recordedBy: Jukka Salmela; Jari Ilmonen; individualCount: 1; sex: female; **Taxon:** scientificName: Dolichopus lancearius; order: Diptera; family: Dolichopodidae; scientificNameAuthorship: Hedström, 1966; **Location:** country: Finland; stateProvince: PPp; municipality: Tervola; locality: Karhakkamaa; verbatimCoordinates: 7346879:3415620; verbatimCoordinateSystem: ykj; decimalLatitude: 66.203; decimalLongitude: 25.123; geodeticDatum: WGS84; coordinatePrecision: 100; **Identification:** identifiedBy: Jere Kahanpää; **Event:** samplingProtocol: Malaise trap; eventDate: 2004-06-28/08-02; **Record Level:** collectionCode: Priv. Coll. J. Kahanpää; basisOfRecord: PreservedSpecimen
- **Occurrence:** recordedBy: Jukka Salmela; Jari Ilmonen; individualCount: 2; sex: 1 male, 1 female; **Taxon:** scientificName: Dolichopus lancearius; order: Diptera; family: Dolichopodidae; scientificNameAuthorship: Hedström, 1966; **Location:** country: Finland; stateProvince: PPp; municipality: Tervola; locality: Karhakkamaanjänkä; verbatimCoordinates: 7346254:3415785; verbatimCoordinateSystem: ykj; decimalLatitude: 66.197; decimalLongitude: 25.127; geodeticDatum: WGS84; coordinatePrecision: 100; **Identification:** identifiedBy: Jere Kahanpää; **Event:** samplingProtocol: Malaise trap; eventDate: 2004-06-28/08-02; **Record Level:** collectionCode: Priv. Coll. J. Kahanpää; basisOfRecord: PreservedSpecimen

#### Distribution

New to Finland. Also known from Sweden, Norway and Asian Russia (Hedström 1966, Grichanov 2006, Negrobov et al. 2010, Pollet 2013).

### Dolichopus nigripes

Fallén, 1823

http://www.faunaeur.org/full_results.php?id=135572

#### Materials

- **Occurrence:** recordedBy: Jere Kahanpää; individualCount: 1; sex: male; **Taxon:** scientificName: Dolichopus nigripes; order: Diptera; family: Dolichopodidae; scientificNameAuthorship: Fallén, 1823; **Location:** country: Finland; stateProvince: U; municipality: Nurmijärvi; locality: Lallinsuo; verbatimCoordinates: 6700928:3372711; verbatimCoordinateSystem: ykj; decimalLatitude: 60.399; decimalLongitude: 24.688; geodeticDatum: WGS84; coordinatePrecision: 50; **Identification:** identifiedBy: Jere Kahanpää; **Event:** samplingProtocol: pan trap (white); eventDate: 2007-05-23/04-07; **Record Level:** collectionCode: Priv. Coll. J. Kahanpää; basisOfRecord: PreservedSpecimen
- **Occurrence:** recordedBy: Jere Kahanpää; individualCount: 2; sex: 1 male, 1 female; **Taxon:** scientificName: Dolichopus nigripes; order: Diptera; family: Dolichopodidae; scientificNameAuthorship: Fallén, 1823; **Location:** country: Finland; stateProvince: V; municipality: Karjaa; locality: Långån; verbatimCoordinates: 6665901:3321021; verbatimCoordinateSystem: ykj; decimalLatitude: 60.066; decimalLongitude: 23.782; geodeticDatum: WGS84; coordinatePrecision: 50; **Identification:** identifiedBy: Jere Kahanpää; **Event:** samplingProtocol: Malaise trap; eventDate: 2007-05-29/07-10; **Record Level:** collectionCode: Priv. Coll. J. Kahanpää; basisOfRecord: PreservedSpecimen

#### Distribution

New to Finland. Widespread in Europe north of the Alps (Pollet 2013).

### Sybistroma discipes

(Germar, 1817)

http://www.faunaeur.org/full_results.php?id=136349

#### Materials

- **Occurrence:** recordedBy: Jussi Koistinen; individualCount: 1; sex: male; otherCatalogNumbers: labelcode:JKo12-00194; **Taxon:** scientificName: Sybistroma discipes; order: Diptera; family: Dolichopodidae; scientificNameAuthorship: (Germar, 1817); **Location:** country: Finland; stateProvince: U; municipality: Espoo; locality: Siikajärvi; verbatimCoordinates: 6686:3363; verbatimCoordinateSystem: ykj; decimalLatitude: 60.27; decimalLongitude: 24.53; geodeticDatum: WGS84; **Identification:** identifiedBy: Jere Kahanpää; **Event:** eventDate: 2012-08-20; **Record Level:** collectionID: http://id.luomus.fi/HR.110; institutionCode: MZH; collectionCode: Diptera Fennica; basisOfRecord: PreservedSpecimen

#### Distribution

New to Finland. Widespread in Europe, also known from the Near East (Pollet 2013).

### Argyra setulipes

Becker, 1918

http://www.faunaeur.org/full_results.php?id=135240

#### Materials

- **Occurrence:** recordedBy: Jere Kahanpää; individualCount: 4; sex: males; **Taxon:** scientificName: Argyra setulipes; order: Diptera; family: Dolichopodidae; scientificNameAuthorship: Becker, 1918; **Location:** country: Finland; stateProvince: U; municipality: Siuntio; locality: Lempansån; verbatimCoordinates: 6681386:3341378; verbatimCoordinateSystem: ykj; decimalLatitude: 60.213; decimalLongitude: 24.135; geodeticDatum: WGS84; coordinatePrecision: 50; **Identification:** identifiedBy: Jere Kahanpää; **Event:** samplingProtocol: Malaise trap; eventDate: 2007-06-08/07-10; **Record Level:** collectionCode: Priv. Coll. J. Kahanpää; basisOfRecord: PreservedSpecimen

#### Biology

Caught on the silt banks of a small stream in a herb-rich forest.

#### Distribution

New to Finland. An east European species, previously recorded from Austria, Hungary, European Russia and Ukraine (Pollet 2013).

### Asyndetus latifrons

(Loew, 1857)

http://www.faunaeur.org/full_results.php?id=135254

#### Materials

- **Occurrence:** recordedBy: Jari Flinck; individualCount: 1; sex: female; otherCatalogNumbers: labelcode: JF10-4448; **Taxon:** scientificName: Asyndetus latifrons; order: Diptera; family: Dolichopodidae; scientificNameAuthorship: (Loew, 1857); **Location:** country: Finland; stateProvince: U; municipality: Loviisa; locality: Kuggom, sand pit; verbatimCoordinates: 67060:84548; verbatimCoordinateSystem: etrs-tm35fin; decimalLatitude: 60.488; decimalLongitude: 26.178; geodeticDatum: WGS84; coordinatePrecision: 200; **Identification:** identifiedBy: Marc Pollet; **Event:** samplingProtocol: sweep netting; eventDate: 2010-07-15; **Record Level:** basisOfRecord: PreservedSpecimen
- **Occurrence:** recordedBy: Jari Flinck; individualCount: 19; sex: 10 males, 9 females; otherCatalogNumbers: labelcode: Tube 11/2; **Taxon:** scientificName: Asyndetus latifrons; order: Diptera; family: Dolichopodidae; scientificNameAuthorship: (Loew, 1857); **Location:** country: Finland; stateProvince: U; municipality: Loviisa; locality: Kuggum, mossen; verbatimCoordinates: 6706975:8454413; verbatimCoordinateSystem: etrs-tm35fin; decimalLatitude: 60.496; decimalLongitude: 26.170; geodeticDatum: WGS84; coordinatePrecision: 200; **Identification:** identifiedBy: Marc Pollet; **Event:** samplingProtocol: Malaise trap; eventDate: 2011-07-1/5; **Record Level:** basisOfRecord: PreservedSpecimen
- **Occurrence:** recordedBy: Jari Flinck; individualCount: 1; sex: male; otherCatalogNumbers: labelcode: JF11-0875; **Taxon:** scientificName: Asyndetus latifrons; order: Diptera; family: Dolichopodidae; scientificNameAuthorship: (Loew, 1857); **Location:** country: Finland; stateProvince: U; municipality: Loviisa; locality: Kuggom, sand pit; verbatimCoordinates: 67060:84548; verbatimCoordinateSystem: etrs-tm35fin; decimalLatitude: 60.488; decimalLongitude: 26.178; geodeticDatum: WGS84; coordinatePrecision: 200; **Identification:** identifiedBy: Stefan Naglis; **Event:** samplingProtocol: pan trap; eventDate: 2011-07-02; **Record Level:** basisOfRecord: PreservedSpecimen
- **Occurrence:** recordedBy: Jari Flinck; individualCount: 1; sex: male; otherCatalogNumbers: labelcode: JF11-0883; **Taxon:** scientificName: Asyndetus latifrons; order: Diptera; family: Dolichopodidae; scientificNameAuthorship: (Loew, 1857); **Location:** country: Finland; stateProvince: U; municipality: Loviisa; locality: Kuggom, sand pit; verbatimCoordinates: 67060:84548; verbatimCoordinateSystem: etrs-tm35fin; decimalLatitude: 60.488; decimalLongitude: 26.178; geodeticDatum: WGS84; coordinatePrecision: 200; **Identification:** identifiedBy: Marc Pollet; **Event:** samplingProtocol: pan trap; eventDate: 2011-07-02; **Record Level:** basisOfRecord: PreservedSpecimen
- **Occurrence:** recordedBy: Jari Flinck; individualCount: 1; sex: female; otherCatalogNumbers: labelcode: Tube 11/1; **Taxon:** scientificName: Asyndetus latifrons; order: Diptera; family: Dolichopodidae; scientificNameAuthorship: (Loew, 1857); **Location:** country: Finland; stateProvince: U; municipality: Loviisa; locality: Kuggom, sand pit; verbatimCoordinates: 67060:84548; verbatimCoordinateSystem: etrs-tm35fin; decimalLatitude: 60.488; decimalLongitude: 26.178; geodeticDatum: WGS84; coordinatePrecision: 200; **Identification:** identifiedBy: Marc Pollet; **Event:** samplingProtocol: pan trap; eventDate: 2011-07-02; **Record Level:** basisOfRecord: PreservedSpecimen
- **Occurrence:** recordedBy: Jari Flinck; individualCount: 1; sex: male; otherCatalogNumbers: labelcode: JF11-0887; **Taxon:** scientificName: Asyndetus latifrons; order: Diptera; family: Dolichopodidae; scientificNameAuthorship: (Loew, 1857); **Location:** country: Finland; stateProvince: U; municipality: Loviisa; locality: Kuggom, sand pit; verbatimCoordinates: 67060:84548; verbatimCoordinateSystem: etrs-tm35fin; decimalLatitude: 60.488; decimalLongitude: 26.178; geodeticDatum: WGS84; coordinatePrecision: 200; **Identification:** identifiedBy: Marc Pollet; **Event:** samplingProtocol: pan trap; eventDate: 2011-07-02; **Record Level:** basisOfRecord: PreservedSpecimen
- **Occurrence:** recordedBy: Jari Flinck; individualCount: 4; sex: 3 males, 1 female; otherCatalogNumbers: labelcode: Tube 11/4; **Taxon:** scientificName: Asyndetus latifrons; order: Diptera; family: Dolichopodidae; scientificNameAuthorship: (Loew, 1857); **Location:** country: Finland; stateProvince: U; municipality: Loviisa; locality: Kuggom, sand pit; verbatimCoordinates: 67060:84548; verbatimCoordinateSystem: etrs-tm35fin; decimalLatitude: 60.488; decimalLongitude: 26.178; geodeticDatum: WGS84; coordinatePrecision: 200; **Identification:** identifiedBy: Marc Pollet; **Event:** samplingProtocol: pan trap; eventDate: 2011-07-06/07; **Record Level:** basisOfRecord: PreservedSpecimen
- **Occurrence:** recordedBy: Jari Flinck; individualCount: 13; sex: 9 males, 4 females; otherCatalogNumbers: labelcode: Tube 11/3; **Taxon:** scientificName: Asyndetus latifrons; order: Diptera; family: Dolichopodidae; scientificNameAuthorship: (Loew, 1857); **Location:** country: Finland; stateProvince: U; municipality: Loviisa; locality: Kuggom, sand pit; verbatimCoordinates: 67060:84548; verbatimCoordinateSystem: etrs-tm35fin; decimalLatitude: 60.488; decimalLongitude: 26.178; geodeticDatum: WGS84; coordinatePrecision: 200; **Identification:** identifiedBy: Marc Pollet; **Event:** samplingProtocol: pan trap; eventDate: 2011-07-08/09; **Record Level:** basisOfRecord: PreservedSpecimen
- **Occurrence:** recordedBy: Jari Flinck; individualCount: 2; sex: females; otherCatalogNumbers: labelcode: Tube 11/5; **Taxon:** scientificName: Asyndetus latifrons; order: Diptera; family: Dolichopodidae; scientificNameAuthorship: (Loew, 1857); **Location:** country: Finland; stateProvince: U; municipality: Loviisa; locality: Kuggom, sand pit; verbatimCoordinates: 67060:84548; verbatimCoordinateSystem: etrs-tm35fin; decimalLatitude: 60.488; decimalLongitude: 26.178; geodeticDatum: WGS84; coordinatePrecision: 200; **Identification:** identifiedBy: Marc Pollet; **Event:** samplingProtocol: pan trap; eventDate: 2011-07-10/11; **Record Level:** basisOfRecord: PreservedSpecimen
- **Occurrence:** recordedBy: Jari Flinck; individualCount: 9; sex: 6 males, 3 females; otherCatalogNumbers: labelcode: Tube 11/6; **Taxon:** scientificName: Asyndetus latifrons; order: Diptera; family: Dolichopodidae; scientificNameAuthorship: (Loew, 1857); **Location:** country: Finland; stateProvince: U; municipality: Loviisa; locality: Trollberget; verbatimCoordinates: 670314:845904; verbatimCoordinateSystem: etrs-tm35fin; decimalLatitude: 60.462; decimalLongitude: 26.255; geodeticDatum: WGS84; coordinatePrecision: 200; **Identification:** identifiedBy: Marc Pollet; **Event:** samplingProtocol: pan trap; eventDate: 2011-07-11/13; **Record Level:** basisOfRecord: PreservedSpecimen
- **Occurrence:** recordedBy: Jari Flinck; individualCount: 3; sex: 2 males, 1 female; otherCatalogNumbers: labelcode: Tube 11/19; **Taxon:** scientificName: Asyndetus latifrons; order: Diptera; family: Dolichopodidae; scientificNameAuthorship: (Loew, 1857); **Location:** country: Finland; stateProvince: U; municipality: Helsinki; locality: Herttoniemi, Fastholma; verbatimCoordinates: 6675935:8390477; verbatimCoordinateSystem: etrs-tm35fin; decimalLatitude: 60.205; decimalLongitude: 25.024; geodeticDatum: WGS84; coordinatePrecision: 200; **Identification:** identifiedBy: Marc Pollet; **Event:** samplingProtocol: Malaise trap; eventDate: 2011-09-10/17; **Record Level:** basisOfRecord: PreservedSpecimen

#### Distribution

New to Finland. Widespread in Europe reaching the Near East and Siberia (Pollet 2013).

### Chrysotus cupreus

(Macquart, 1827)

http://www.faunaeur.org/full_results.php?id=135341

#### Materials

- **Occurrence:** recordedBy: Jere Kahanpää; individualCount: 3; sex: 2 males, 1 female; otherCatalogNumbers: labelcode: jka07-01767, jka07-01794, jka07-01766; **Taxon:** scientificName: Chrysotus cupreus; order: Diptera; family: Dolichopodidae; scientificNameAuthorship: (Macquart, 1827); **Location:** country: Finland; stateProvince: A; municipality: Lemland; locality: Näsen; verbatimCoordinates: 66751:31171; verbatimCoordinateSystem: ykj; decimalLatitude: 60.009; decimalLongitude: 20.126; geodeticDatum: WGS84; coordinatePrecision: 300; **Identification:** identifiedBy: Jere Kahanpää; **Event:** eventDate: 2007-06-15; **Record Level:** collectionCode: Priv. Coll. J. Kahanpää; basisOfRecord: PreservedSpecimen

#### Distribution

New to Finland. Widespread in Europe, reaching the Middle East (Pollet 2013).

### Campsicnemus femoratus

Ringdahl, 1949

http://www.faunaeur.org/full_results.php?id=135291

#### Materials

- **Occurrence:** recordedBy: Jukka Salmela; individualCount: 5; sex: 4 males, 1 female; **Taxon:** scientificName: Campsicnemus femoratus; order: Diptera; family: Dolichopodidae; scientificNameAuthorship: Ringdahl, 1949; **Location:** country: Finland; stateProvince: Ks; municipality: Kuusamo; locality: Paahtojärvi; verbatimCoordinates: 7347004:3614371; verbatimCoordinateSystem: ykj; decimalLatitude: 66.195; decimalLongitude: 29.535; geodeticDatum: WGS84; coordinatePrecision: 100; **Identification:** identifiedBy: Jere Kahanpää; **Event:** samplingProtocol: Malaise trap; eventDate: 2005-05-23/06-20; **Record Level:** collectionCode: Priv. Coll. J. Kahanpää; basisOfRecord: PreservedSpecimen
- **Occurrence:** recordedBy: Jukka Salmela; individualCount: 1; sex: female; **Taxon:** scientificName: Campsicnemus femoratus; order: Diptera; family: Dolichopodidae; scientificNameAuthorship: Ringdahl, 1949; **Location:** country: Finland; stateProvince: Ks; municipality: Kuusamo; locality: Isojärvenpuro; verbatimCoordinates: 7357735:3608139; verbatimCoordinateSystem: ykj; decimalLatitude: 66.293; decimalLongitude: 29.406; geodeticDatum: WGS84; coordinatePrecision: 100; **Identification:** identifiedBy: Jere Kahanpää; **Event:** samplingProtocol: Malaise trap; eventDate: 2005-05-23/06-20; **Record Level:** collectionCode: Priv. Coll. J. Kahanpää; basisOfRecord: PreservedSpecimen
- **Occurrence:** recordedBy: Jukka Salmela; individualCount: 1; sex: female; **Taxon:** scientificName: Campsicnemus femoratus; order: Diptera; family: Dolichopodidae; scientificNameAuthorship: Ringdahl, 1949; **Location:** country: Finland; stateProvince: Ks; municipality: Kuusamo; locality: Saaripuro; verbatimCoordinates: 7357336:3611517; verbatimCoordinateSystem: ykj; decimalLatitude: 66.288; decimalLongitude: 29.481; geodeticDatum: WGS84; coordinatePrecision: 100; **Identification:** identifiedBy: Jere Kahanpää; **Event:** samplingProtocol: Malaise trap; eventDate: 2005-05-23/06-20; **Record Level:** collectionCode: Priv. Coll. J. Kahanpää; basisOfRecord: PreservedSpecimen
- **Occurrence:** recordedBy: Jukka Salmela; individualCount: 3; sex: 1 male, 2 females; **Taxon:** scientificName: Campsicnemus femoratus; order: Diptera; family: Dolichopodidae; scientificNameAuthorship: Ringdahl, 1949; **Location:** country: Finland; stateProvince: Ks; municipality: Kuusamo; locality: Uopajanpuro; verbatimCoordinates: 7362617:3612763; verbatimCoordinateSystem: ykj; decimalLatitude: 66.335; decimalLongitude: 29.513; geodeticDatum: WGS84; coordinatePrecision: 100; **Identification:** identifiedBy: Jere Kahanpää; **Event:** samplingProtocol: Malaise trap; eventDate: 2005-05-23/06-20; **Record Level:** collectionCode: Priv. Coll. J. Kahanpää; basisOfRecord: PreservedSpecimen
- **Occurrence:** recordedBy: Jukka Salmela; individualCount: 3; sex: males; **Taxon:** scientificName: Campsicnemus femoratus; order: Diptera; family: Dolichopodidae; scientificNameAuthorship: Ringdahl, 1949; **Location:** country: Finland; stateProvince: Ks; municipality: Kuusamo; locality: Uopaja; verbatimCoordinates: 7363447:3613209; verbatimCoordinateSystem: ykj; decimalLatitude: 66.343; decimalLongitude: 29.524; geodeticDatum: WGS84; coordinatePrecision: 100; **Identification:** identifiedBy: Jere Kahanpää; **Event:** samplingProtocol: Malaise trap; eventDate: 2005-05-23/06-20; **Record Level:** collectionCode: Priv. Coll. J. Kahanpää; basisOfRecord: PreservedSpecimen
- **Occurrence:** recordedBy: Jukka Salmela; individualCount: 1; sex: male; **Taxon:** scientificName: Campsicnemus femoratus; order: Diptera; family: Dolichopodidae; scientificNameAuthorship: Ringdahl, 1949; **Location:** country: Finland; stateProvince: Ks; municipality: Kuusamo; locality: Merenoja; verbatimCoordinates: 7364088:3605383; verbatimCoordinateSystem: ykj; decimalLatitude: 66.351; decimalLongitude: 29.350; geodeticDatum: WGS84; coordinatePrecision: 100; **Identification:** identifiedBy: Jere Kahanpää; **Event:** samplingProtocol: Malaise trap; eventDate: 2005-05-23/06-20; **Record Level:** collectionCode: Priv. Coll. J. Kahanpää; basisOfRecord: PreservedSpecimen
- **Occurrence:** recordedBy: Jukka Salmela; individualCount: 2; sex: males; **Taxon:** scientificName: Campsicnemus femoratus; order: Diptera; family: Dolichopodidae; scientificNameAuthorship: Ringdahl, 1949; **Location:** country: Finland; stateProvince: Ks; municipality: Kuusamo; locality: Kalliojoki; verbatimCoordinates: 7344909:3610594; verbatimCoordinateSystem: ykj; decimalLatitude: 66.177; decimalLongitude: 29.449; geodeticDatum: WGS84; coordinatePrecision: 100; **Identification:** identifiedBy: Jere Kahanpää; **Event:** samplingProtocol: Malaise trap; eventDate: 2005-05-23/06-20; **Record Level:** collectionCode: Priv. Coll. J. Kahanpää; basisOfRecord: PreservedSpecimen
- **Occurrence:** recordedBy: Jukka Salmela; individualCount: 2; sex: males; **Taxon:** scientificName: Campsicnemus femoratus; order: Diptera; family: Dolichopodidae; scientificNameAuthorship: Ringdahl, 1949; **Location:** country: Finland; stateProvince: Ks; municipality: Kuusamo; locality: Putaanoja; verbatimCoordinates: 7367392:3608548; verbatimCoordinateSystem: ykj; decimalLatitude: 66.380; decimalLongitude: 29.423; geodeticDatum: WGS84; coordinatePrecision: 100; **Identification:** identifiedBy: Jere Kahanpää; **Event:** samplingProtocol: Malaise trap; eventDate: 2005-05-23/06-20; **Record Level:** collectionCode: Priv. Coll. J. Kahanpää; basisOfRecord: PreservedSpecimen
- **Occurrence:** recordedBy: Jukka Salmela; individualCount: 2; sex: males; **Taxon:** scientificName: Campsicnemus femoratus; order: Diptera; family: Dolichopodidae; scientificNameAuthorship: Ringdahl, 1949; **Location:** country: Finland; stateProvince: Ks; municipality: Kuusamo; locality: Uopaja; verbatimCoordinates: 7363447:3613209; verbatimCoordinateSystem: ykj; decimalLatitude: 66.343; decimalLongitude: 29.524; geodeticDatum: WGS84; coordinatePrecision: 100; **Identification:** identifiedBy: Jere Kahanpää; **Event:** samplingProtocol: Malaise trap; eventDate: 2005-05-23/06-20; **Record Level:** collectionCode: Priv. Coll. J. Kahanpää; basisOfRecord: PreservedSpecimen
- **Occurrence:** recordedBy: Jukka Salmela; individualCount: 1; sex: male; **Taxon:** scientificName: Campsicnemus femoratus; order: Diptera; family: Dolichopodidae; scientificNameAuthorship: Ringdahl, 1949; **Location:** country: Finland; stateProvince: Ks; municipality: Taivalkoski; locality: Paavonoja; verbatimCoordinates: 7295643:3565726; verbatimCoordinateSystem: ykj; decimalLatitude: 65.748; decimalLongitude: 28.430; geodeticDatum: WGS84; coordinatePrecision: 100; **Identification:** identifiedBy: Jere Kahanpää; **Event:** samplingProtocol: Malaise trap; eventDate: 2005-08-03/09/20; **Record Level:** collectionCode: Priv. Coll. J. Kahanpää; basisOfRecord: PreservedSpecimen
- **Occurrence:** recordedBy: Jukka Salmela; individualCount: 13; sex: 2 males, 11 females; **Taxon:** scientificName: Campsicnemus femoratus; order: Diptera; family: Dolichopodidae; scientificNameAuthorship: Ringdahl, 1949; **Location:** country: Finland; stateProvince: Ks; municipality: Taivalkoski; locality: Hevosniitynoja; verbatimCoordinates: 7268926:3555642; verbatimCoordinateSystem: ykj; decimalLatitude: 65.511; decimalLongitude: 28.199; geodeticDatum: WGS84; coordinatePrecision: 100; **Identification:** identifiedBy: Jere Kahanpää; **Event:** samplingProtocol: Malaise trap; eventDate: 2006-05-31/07-03; **Record Level:** collectionCode: Priv. Coll. J. Kahanpää; basisOfRecord: PreservedSpecimen
- **Occurrence:** recordedBy: Jukka Salmela; individualCount: 1; sex: female; **Taxon:** scientificName: Campsicnemus femoratus; order: Diptera; family: Dolichopodidae; scientificNameAuthorship: Ringdahl, 1949; **Location:** country: Finland; stateProvince: Ks; municipality: Taivalkoski; locality: Horsmanoja; verbatimCoordinates: 7246144:3558778; verbatimCoordinateSystem: ykj; decimalLatitude: 65.306; decimalLongitude: 28.257; geodeticDatum: WGS84; coordinatePrecision: 100; **Identification:** identifiedBy: Jere Kahanpää; **Event:** samplingProtocol: Malaise trap; eventDate: 2006-05-31/07-03; **Record Level:** collectionCode: Priv. Coll. J. Kahanpää; basisOfRecord: PreservedSpecimen
- **Occurrence:** recordedBy: Jukka Salmela; individualCount: 1; sex: female; **Taxon:** scientificName: Campsicnemus femoratus; order: Diptera; family: Dolichopodidae; scientificNameAuthorship: Ringdahl, 1949; **Location:** country: Finland; stateProvince: Ks; municipality: Taivalkoski; locality: Syväoja; verbatimCoordinates: 7299627:3560581; verbatimCoordinateSystem: ykj; decimalLatitude: 65.785; decimalLongitude: 28.319; geodeticDatum: WGS84; coordinatePrecision: 100; **Identification:** identifiedBy: Jere Kahanpää; **Event:** samplingProtocol: Malaise trap; eventDate: 2006-05-31/07-03; **Record Level:** collectionCode: Priv. Coll. J. Kahanpää; basisOfRecord: PreservedSpecimen
- **Occurrence:** recordedBy: Jukka Salmela; individualCount: 1; sex: male; **Taxon:** scientificName: Campsicnemus femoratus; order: Diptera; family: Dolichopodidae; scientificNameAuthorship: Ringdahl, 1949; **Location:** country: Finland; stateProvince: Ks; municipality: Taivalkoski; locality: Säkkisenoja; verbatimCoordinates: 7280237:3534568; verbatimCoordinateSystem: ykj; decimalLatitude: 65.615; decimalLongitude: 27.746; geodeticDatum: WGS84; coordinatePrecision: 100; **Identification:** identifiedBy: Jere Kahanpää; **Event:** samplingProtocol: Malaise trap; eventDate: 2006-05-31/07-03; **Record Level:** collectionCode: Priv. Coll. J. Kahanpää; basisOfRecord: PreservedSpecimen
- **Occurrence:** recordedBy: Jukka Salmela; individualCount: 1; sex: male; **Taxon:** scientificName: Campsicnemus femoratus; order: Diptera; family: Dolichopodidae; scientificNameAuthorship: Ringdahl, 1949; **Location:** country: Finland; stateProvince: Ks; municipality: Taivalkoski; locality: Pahkaoja; verbatimCoordinates: 7281402:3560358; verbatimCoordinateSystem: ykj; decimalLatitude: 65.622; decimalLongitude: 28.306; geodeticDatum: WGS84; coordinatePrecision: 100; **Identification:** identifiedBy: Jere Kahanpää; **Event:** samplingProtocol: Malaise trap; eventDate: 2006-05-31/07-03; **Record Level:** collectionCode: Priv. Coll. J. Kahanpää; basisOfRecord: PreservedSpecimen
- **Occurrence:** recordedBy: Jukka Salmela; individualCount: 1; sex: female; **Taxon:** scientificName: Campsicnemus femoratus; order: Diptera; family: Dolichopodidae; scientificNameAuthorship: Ringdahl, 1949; **Location:** country: Finland; stateProvince: Ks; municipality: Taivalkoski; locality: Hurunoja; verbatimCoordinates: 7248770:3567830; verbatimCoordinateSystem: ykj; decimalLatitude: 65.328; decimalLongitude: 28.452; geodeticDatum: WGS84; coordinatePrecision: 100; **Identification:** identifiedBy: Jere Kahanpää; **Event:** samplingProtocol: Malaise trap; eventDate: 2006-05-31/07-03; **Record Level:** collectionCode: Priv. Coll. J. Kahanpää; basisOfRecord: PreservedSpecimen
- **Occurrence:** recordedBy: Jukka Salmela; individualCount: 1; sex: male; **Taxon:** scientificName: Campsicnemus femoratus; order: Diptera; family: Dolichopodidae; scientificNameAuthorship: Ringdahl, 1949; **Location:** country: Finland; stateProvince: Ks; municipality: Taivalkoski; locality: Pajuoja; verbatimCoordinates: 7254119:3564978; verbatimCoordinateSystem: ykj; decimalLatitude: 65.376; decimalLongitude: 28.393; geodeticDatum: WGS84; coordinatePrecision: 100; **Identification:** identifiedBy: Jere Kahanpää; **Event:** samplingProtocol: Malaise trap; eventDate: 2006-05-31/07-03; **Record Level:** collectionCode: Priv. Coll. J. Kahanpää; basisOfRecord: PreservedSpecimen
- **Occurrence:** recordedBy: Jukka Salmela; individualCount: 6; sex: 1 male, 5 females; **Taxon:** scientificName: Campsicnemus femoratus; order: Diptera; family: Dolichopodidae; scientificNameAuthorship: Ringdahl, 1949; **Location:** country: Finland; stateProvince: Ks; municipality: Taivalkoski; locality: Kylmäoja; verbatimCoordinates: 7275293:3554865; verbatimCoordinateSystem: ykj; decimalLatitude: 65.568; decimalLongitude: 28.185; geodeticDatum: WGS84; coordinatePrecision: 100; **Identification:** identifiedBy: Jere Kahanpää; **Event:** samplingProtocol: Malaise trap; eventDate: 2006-05-31/07-03; **Record Level:** collectionCode: Priv. Coll. J. Kahanpää; basisOfRecord: PreservedSpecimen
- **Occurrence:** recordedBy: Jukka Salmela; individualCount: 2; sex: 1 male, 1 female; **Taxon:** scientificName: Campsicnemus femoratus; order: Diptera; family: Dolichopodidae; scientificNameAuthorship: Ringdahl, 1949; **Location:** country: Finland; stateProvince: Ks; municipality: Taivalkoski; locality: Hevosniitynoja; verbatimCoordinates: 7268926:3555642; verbatimCoordinateSystem: ykj; decimalLatitude: 65.511; decimalLongitude: 28.199; geodeticDatum: WGS84; coordinatePrecision: 100; **Identification:** identifiedBy: Jere Kahanpää; **Event:** samplingProtocol: Malaise trap; eventDate: 2006-08-1/09-15; **Record Level:** collectionCode: Priv. Coll. J. Kahanpää; basisOfRecord: PreservedSpecimen
- **Occurrence:** recordedBy: Jukka Salmela; individualCount: 1; sex: male; **Taxon:** scientificName: Campsicnemus femoratus; order: Diptera; family: Dolichopodidae; scientificNameAuthorship: Ringdahl, 1949; **Location:** country: Finland; stateProvince: Ks; municipality: Taivalkoski; locality: Syväoja; verbatimCoordinates: 7299627:3560581; verbatimCoordinateSystem: ykj; decimalLatitude: 65.785; decimalLongitude: 28.319; geodeticDatum: WGS84; coordinatePrecision: 100; **Identification:** identifiedBy: Jere Kahanpää; **Event:** samplingProtocol: Malaise trap; eventDate: 2006-08-1/09-15; **Record Level:** collectionCode: Priv. Coll. J. Kahanpää; basisOfRecord: PreservedSpecimen
- **Occurrence:** recordedBy: Jukka Salmela; individualCount: 1; sex: male; **Taxon:** scientificName: Campsicnemus femoratus; order: Diptera; family: Dolichopodidae; scientificNameAuthorship: Ringdahl, 1949; **Location:** country: Finland; stateProvince: PPe; municipality: Pudasjärvi; locality: Pelto-oja; verbatimCoordinates: 7243241:3547649; verbatimCoordinateSystem: ykj; decimalLatitude: 65.282; decimalLongitude: 28.017; geodeticDatum: WGS84; coordinatePrecision: 100; **Identification:** identifiedBy: Jere Kahanpää; **Event:** samplingProtocol: Malaise trap; eventDate: 2006-08-1/09-15; **Record Level:** collectionCode: Priv. Coll. J. Kahanpää; basisOfRecord: PreservedSpecimen
- **Occurrence:** recordedBy: Jukka Salmela; Jari Ilmonen; individualCount: 1; sex: male; **Taxon:** scientificName: Campsicnemus femoratus; order: Diptera; family: Dolichopodidae; scientificNameAuthorship: Ringdahl, 1949; **Location:** country: Finland; stateProvince: PPp; municipality: Tervola; locality: Yrttijänkä; verbatimCoordinates: 7346833:3407825; verbatimCoordinateSystem: ykj; decimalLatitude: 66.200; decimalLongitude: 24.950; geodeticDatum: WGS84; coordinatePrecision: 100; **Identification:** identifiedBy: Jere Kahanpää; **Event:** samplingProtocol: Malaise trap; eventDate: 2004-05-29/06-28; **Record Level:** collectionCode: Priv. Coll. J. Kahanpää; basisOfRecord: PreservedSpecimen
- **Occurrence:** recordedBy: Jukka Salmela; Jari Ilmonen; individualCount: 1; sex: male; **Taxon:** scientificName: Campsicnemus femoratus; order: Diptera; family: Dolichopodidae; scientificNameAuthorship: Ringdahl, 1949; **Location:** country: Finland; stateProvince: PPp; municipality: Tervola; locality: Piilola; verbatimCoordinates: 7347548:3406930; verbatimCoordinateSystem: ykj; decimalLatitude: 66.207; decimalLongitude: 24.929; geodeticDatum: WGS84; coordinatePrecision: 100; **Identification:** identifiedBy: Jere Kahanpää; **Event:** samplingProtocol: Malaise trap; eventDate: 2004-05-29/06-28; **Record Level:** collectionCode: Priv. Coll. J. Kahanpää; basisOfRecord: PreservedSpecimen
- **Occurrence:** recordedBy: Jukka Salmela; individualCount: 4; sex: female; **Taxon:** scientificName: Campsicnemus femoratus; order: Diptera; family: Dolichopodidae; scientificNameAuthorship: Ringdahl, 1949; **Location:** country: Finland; stateProvince: Kn; municipality: Suomussalmi; locality: Toskanoja; verbatimCoordinates: 7237249:3555026; verbatimCoordinateSystem: ykj; decimalLatitude: 65.227; decimalLongitude: 28.173; geodeticDatum: WGS84; coordinatePrecision: 100; **Identification:** identifiedBy: Jere Kahanpää; **Event:** samplingProtocol: Malaise trap; eventDate: 2006-05-31/07-03; **Record Level:** collectionCode: Priv. Coll. J. Kahanpää; basisOfRecord: PreservedSpecimen
- **Occurrence:** recordedBy: Jukka Salmela; individualCount: 11; sex: female; **Taxon:** scientificName: Campsicnemus femoratus; order: Diptera; family: Dolichopodidae; scientificNameAuthorship: Ringdahl, 1949; **Location:** country: Finland; stateProvince: Kn; municipality: Puolanka; locality: Paljakkaoja; verbatimCoordinates: 7233015:3551696; verbatimCoordinateSystem: ykj; decimalLatitude: 65.189; decimalLongitude: 28.100; geodeticDatum: WGS84; coordinatePrecision: 100; **Identification:** identifiedBy: Jere Kahanpää; **Event:** samplingProtocol: Malaise trap; eventDate: 2006-05-31/07-03; **Record Level:** collectionCode: Priv. Coll. J. Kahanpää; basisOfRecord: PreservedSpecimen

#### Biology

*Campsicnemus femoratus* is common and occasionally abundant along forest streams in the central boreal zone of Finland. Its rarity in museum collections is explained by the combination of a northern distribution, an early/late occurrence of the adults and a species-poor habitat often skipped by collectors of Dolichopodidae: the adults are on the wing in May and September when these forests may still have a partial snow cover.

#### Distribution

New to Finland. Previously known from Sweden and Russian East Siberia (Negrobov 1991, Ringdahl 1949).

### Micromorphus claripennis

(Strobl, 1899)

http://www.faunaeur.org/full_results.php?id=136047

#### Materials

- **Occurrence:** recordedBy: Jere Kahanpää; individualCount: 1; sex: male; **Taxon:** scientificName: Micromorphus claripennis; order: Diptera; family: Dolichopodidae; scientificNameAuthorship: (Strobl, 1899); **Location:** country: Finland; stateProvince: V; municipality: Karjaa; locality: Mustio; verbatimCoordinates: 667368:332560; verbatimCoordinateSystem: ykj; decimalLatitude: 60.138; decimalLongitude: 23.858; geodeticDatum: WGS84; coordinatePrecision: 50; **Identification:** identifiedBy: Jere Kahanpää; **Event:** samplingProtocol: sweep netting; eventDate: 2006-6-20; **Record Level:** collectionID: http://id.luomus.fi/HR.110; institutionCode: MZH; collectionCode: Diptera Fennica; basisOfRecord: PreservedSpecimen
- **Occurrence:** recordedBy: Jere Kahanpää; individualCount: 1; sex: male; **Taxon:** scientificName: Micromorphus claripennis; order: Diptera; family: Dolichopodidae; scientificNameAuthorship: (Strobl, 1899); **Location:** country: Finland; stateProvince: V; municipality: Karjaa; locality: Mustio; verbatimCoordinates: 667368:332560; verbatimCoordinateSystem: ykj; decimalLatitude: 60.138; decimalLongitude: 23.858; geodeticDatum: WGS84; coordinatePrecision: 50; **Identification:** identifiedBy: Jere Kahanpää; **Event:** samplingProtocol: sweep netting; eventDate: 2006-6-20; **Record Level:** collectionID: http://id.luomus.fi/HR.110; institutionCode: MZH; collectionCode: Diptera Fennica; basisOfRecord: PreservedSpecimen
- **Occurrence:** recordedBy: Jere Kahanpää; individualCount: 1; sex: female; **Taxon:** scientificName: Micromorphus claripennis; order: Diptera; family: Dolichopodidae; scientificNameAuthorship: (Strobl, 1899); **Location:** country: Finland; stateProvince: V; municipality: Karjaa; locality: Mustio; verbatimCoordinates: 667368:332560; verbatimCoordinateSystem: ykj; decimalLatitude: 60.138; decimalLongitude: 23.858; geodeticDatum: WGS84; coordinatePrecision: 50; **Identification:** identifiedBy: Jere Kahanpää; **Event:** samplingProtocol: sweep netting; eventDate: 2006-6-20; **Record Level:** collectionID: http://id.luomus.fi/HR.110; institutionCode: MZH; collectionCode: Diptera Fennica; basisOfRecord: PreservedSpecimen
- **Occurrence:** recordedBy: Jere Kahanpää; individualCount: 1; sex: female; **Taxon:** scientificName: Micromorphus claripennis; order: Diptera; family: Dolichopodidae; scientificNameAuthorship: (Strobl, 1899); **Location:** country: Finland; stateProvince: V; municipality: Karjaa; locality: Mustio; verbatimCoordinates: 667368:332560; verbatimCoordinateSystem: ykj; decimalLatitude: 60.138; decimalLongitude: 23.858; geodeticDatum: WGS84; coordinatePrecision: 50; **Identification:** identifiedBy: Jere Kahanpää; **Event:** samplingProtocol: sweep netting; eventDate: 2006-6-20; **Record Level:** collectionID: http://id.luomus.fi/HR.110; institutionCode: MZH; collectionCode: Diptera Fennica; basisOfRecord: PreservedSpecimen

#### Distribution

New to Finland. Previously known from NW Russia, Germany and Spain (Bellstedt et al. 1999, Carles-Tolrá Hjort-Andersen 2002, Kahanpää and Winqvist 2005). As the European *Micromorphus* are in need of revision, the distribution of most species is poorly known.

#### Notes

Identified using Negrobov (2000).

### Achalcus nigropunctatus

Pollét & Brunhues, 1996

http://www.faunaeur.org/full_results.php?id=135164

#### Materials

- **Occurrence:** recordedBy: Jere Kahanpää; individualCount: 20; sex: 9 males, 11 females; **Taxon:** scientificName: Achalcus nigropunctatus; order: Diptera; family: Dolichopodidae; scientificNameAuthorship: Pollét & Brunhues, 1996; **Location:** country: Finland; stateProvince: V; municipality: Karjaa; locality: Långån; verbatimCoordinates: 6665901:3321021; verbatimCoordinateSystem: ykj; decimalLatitude: 60.066; decimalLongitude: 23.782; geodeticDatum: WGS84; coordinatePrecision: 200; **Identification:** identifiedBy: Jere Kahanpää; **Event:** samplingProtocol: Malaise trap; eventDate: 2007-05-59/07-10; **Record Level:** collectionCode: Priv. Coll. J. Kahanpää; basisOfRecord: PreservedSpecimen
- **Occurrence:** recordedBy: Jere Kahanpää; individualCount: 8; sex: 4 males, 4 females; **Taxon:** scientificName: Achalcus nigropunctatus; order: Diptera; family: Dolichopodidae; scientificNameAuthorship: Pollét & Brunhues, 1996; **Location:** country: Finland; stateProvince: V; municipality: Karjaa; locality: Långån; verbatimCoordinates: 6665901:3321021; verbatimCoordinateSystem: ykj; decimalLatitude: 60.066; decimalLongitude: 23.782; geodeticDatum: WGS84; coordinatePrecision: 200; **Identification:** identifiedBy: Jere Kahanpää; **Event:** samplingProtocol: Malaise trap; eventDate: 2007-07-10/08-04; **Record Level:** collectionCode: Priv. Coll. J. Kahanpää; basisOfRecord: PreservedSpecimen
- **Occurrence:** recordedBy: Olli Autio; Jukka Salmela; individualCount: 1; sex: male; **Taxon:** scientificName: Achalcus nigropunctatus; order: Diptera; family: Dolichopodidae; scientificNameAuthorship: Pollét & Brunhues, 1996; **Location:** country: Finland; stateProvince: A; municipality: Eckerö; locality: Holmträsket; verbatimCoordinates: 670517:309246; verbatimCoordinateSystem: ykj; decimalLatitude: 60.253; decimalLongitude: 19.627; geodeticDatum: WGS84; coordinatePrecision: 200; **Identification:** identifiedBy: Jere Kahanpää; **Event:** samplingProtocol: Malaise trap; eventDate: 2007-06-15/07-27; **Record Level:** collectionCode: Priv. Coll. J. Kahanpää; basisOfRecord: PreservedSpecimen
- **Occurrence:** recordedBy: Olli Autio; Jukka Salmela; individualCount: 9; sex: 5 males, 4 females; **Taxon:** scientificName: Achalcus nigropunctatus; order: Diptera; family: Dolichopodidae; scientificNameAuthorship: Pollét & Brunhues, 1996; **Location:** country: Finland; stateProvince: A; municipality: Jomala; locality: Timmermyran; verbatimCoordinates: 668995:309847; verbatimCoordinateSystem: ykj; decimalLatitude: 60.123; decimalLongitude: 19.765; geodeticDatum: WGS84; coordinatePrecision: 200; **Identification:** identifiedBy: Jere Kahanpää; **Event:** samplingProtocol: Malaise trap; eventDate: 2007-06-15/07-27; **Record Level:** collectionCode: Priv. Coll. J. Kahanpää; basisOfRecord: PreservedSpecimen
- **Occurrence:** recordedBy: Olli Autio; Jukka Salmela; individualCount: 1; sex: male; **Taxon:** scientificName: Achalcus nigropunctatus; order: Diptera; family: Dolichopodidae; scientificNameAuthorship: Pollét & Brunhues, 1996; **Location:** country: Finland; stateProvince: A; municipality: Jomala; locality: Moren; verbatimCoordinates: 669462:310386; verbatimCoordinateSystem: ykj; decimalLatitude: 60.170; decimalLongitude: 19.852; geodeticDatum: WGS84; coordinatePrecision: 200; **Identification:** identifiedBy: Jere Kahanpää; **Event:** samplingProtocol: Malaise trap; eventDate: 2007-06-15/07-27; **Record Level:** collectionCode: Priv. Coll. J. Kahanpää; basisOfRecord: PreservedSpecimen
- **Occurrence:** recordedBy: Olli Autio; Jukka Salmela; individualCount: 2; sex: males; **Taxon:** scientificName: Achalcus nigropunctatus; order: Diptera; family: Dolichopodidae; scientificNameAuthorship: Pollét & Brunhues, 1996; **Location:** country: Finland; stateProvince: A; municipality: Hammarland; locality: Ängesjö; verbatimCoordinates: 670718:309841; verbatimCoordinateSystem: ykj; decimalLatitude: 60.276; decimalLongitude: 19.730; geodeticDatum: WGS84; coordinatePrecision: 200; **Identification:** identifiedBy: Jere Kahanpää; **Event:** samplingProtocol: Malaise trap; eventDate: 2007-06-16/07-28; **Record Level:** collectionCode: Priv. Coll. J. Kahanpää; basisOfRecord: PreservedSpecimen
- **Occurrence:** recordedBy: Jukka Salmela; individualCount: 2; sex: males; **Taxon:** scientificName: Achalcus nigropunctatus; order: Diptera; family: Dolichopodidae; scientificNameAuthorship: Pollét & Brunhues, 1996; **Location:** country: Finland; stateProvince: InL; municipality: Inari; locality: Mustajuurakkojärveen laskeva puro; verbatimCoordinates: 7619338:3554361; verbatimCoordinateSystem: ykj; decimalLatitude: 68.652; decimalLongitude: 28.333; geodeticDatum: WGS84; coordinatePrecision: 200; **Identification:** identifiedBy: Jere Kahanpää; **Event:** samplingProtocol: Malaise trap; eventDate: 2004-07-06/08-01; **Record Level:** collectionCode: Priv. Coll. J. Kahanpää; basisOfRecord: PreservedSpecimen
- **Occurrence:** recordedBy: Jukka Salmela; individualCount: 1; sex: males; **Taxon:** scientificName: Achalcus nigropunctatus; order: Diptera; family: Dolichopodidae; scientificNameAuthorship: Pollét & Brunhues, 1996; **Location:** country: Finland; stateProvince: InL; municipality: Inari; locality: Mustajuurakkojärveen laskeva puro; verbatimCoordinates: 7619338:3554361; verbatimCoordinateSystem: ykj; decimalLatitude: 68.652; decimalLongitude: 28.333; geodeticDatum: WGS84; coordinatePrecision: 200; **Identification:** identifiedBy: Jere Kahanpää; **Event:** samplingProtocol: Malaise trap; eventDate: 2004-07-06/08-01; **Record Level:** collectionCode: Priv. Coll. J. Kahanpää; basisOfRecord: PreservedSpecimen
- **Occurrence:** recordedBy: Jere Kahanpää; individualCount: 3; sex: 1 male, 2 females; **Taxon:** scientificName: Achalcus nigropunctatus; order: Diptera; family: Dolichopodidae; scientificNameAuthorship: Pollét & Brunhues, 1996; **Location:** country: Finland; stateProvince: EP; municipality: Närpiö; locality: Hinjärvsträsket; verbatimCoordinates: 696456:320987; verbatimCoordinateSystem: ykj; decimalLatitude: 62.670; decimalLongitude: 21.330; geodeticDatum: WGS84; coordinatePrecision: 200; **Identification:** identifiedBy: Jere Kahanpää; **Event:** samplingProtocol: Malaise trap; eventDate: 2005-07-01/27; **Record Level:** collectionCode: Priv. Coll. J. Kahanpää; basisOfRecord: PreservedSpecimen
- **Occurrence:** recordedBy: Jere Kahanpää; individualCount: 4; sex: 2 males, 2 females; **Taxon:** scientificName: Achalcus nigropunctatus; order: Diptera; family: Dolichopodidae; scientificNameAuthorship: Pollét & Brunhues, 1996; **Location:** country: Finland; stateProvince: PPe; municipality: Liminka; locality: Virkkula; verbatimCoordinates: 719323:341985; verbatimCoordinateSystem: ykj; decimalLatitude: 64.827; decimalLongitude: 25.308; geodeticDatum: WGS84; coordinatePrecision: 200; **Identification:** identifiedBy: Jere Kahanpää; **Event:** samplingProtocol: Malaise trap; eventDate: 2005-07-04/30; **Record Level:** collectionCode: Priv. Coll. J. Kahanpää; basisOfRecord: PreservedSpecimen
- **Occurrence:** recordedBy: Jukka Salmela; Jari Ilmonen; individualCount: 2; sex: 1 male, 1 female; **Taxon:** scientificName: Achalcus nigropunctatus; order: Diptera; family: Dolichopodidae; scientificNameAuthorship: Pollét & Brunhues, 1996; **Location:** country: Finland; stateProvince: PPp; municipality: Tervola; locality: Keskipalo; verbatimCoordinates: 7344806:3415122; verbatimCoordinateSystem: ykj; decimalLatitude: 66.184; decimalLongitude: 25.113; geodeticDatum: WGS84; coordinatePrecision: 200; **Identification:** identifiedBy: Jere Kahanpää; **Event:** samplingProtocol: Malaise trap; eventDate: 2004-06-28/08-02; **Record Level:** collectionCode: Priv. Coll. J. Kahanpää; basisOfRecord: PreservedSpecimen
- **Occurrence:** recordedBy: Jukka Salmela; Jari Ilmonen; individualCount: 1; sex: male; **Taxon:** scientificName: Achalcus nigropunctatus; order: Diptera; family: Dolichopodidae; scientificNameAuthorship: Pollét & Brunhues, 1996; **Location:** country: Finland; stateProvince: PPp; municipality: Tervola; locality: RuuttulampiIII; verbatimCoordinates: 7347734:3409699; verbatimCoordinateSystem: ykj; decimalLatitude: 66.209; decimalLongitude: 24.991; geodeticDatum: WGS84; coordinatePrecision: 200; **Identification:** identifiedBy: Jere Kahanpää; **Event:** samplingProtocol: Malaise trap; eventDate: 2004-06-28/08-02; **Record Level:** collectionCode: Priv. Coll. J. Kahanpää; basisOfRecord: PreservedSpecimen
- **Occurrence:** recordedBy: Jukka Salmela; Jari Ilmonen; individualCount: 1; sex: male; **Taxon:** scientificName: Achalcus nigropunctatus; order: Diptera; family: Dolichopodidae; scientificNameAuthorship: Pollét & Brunhues, 1996; **Location:** country: Finland; stateProvince: PPp; municipality: Tervola; locality: Sompujärvi NE2; verbatimCoordinates: 7320408:3414913; verbatimCoordinateSystem: ykj; decimalLatitude: 65.966; decimalLongitude: 25.125; geodeticDatum: WGS84; coordinatePrecision: 200; **Identification:** identifiedBy: Jere Kahanpää; **Event:** samplingProtocol: Malaise trap; eventDate: 2004-06-28/08-02; **Record Level:** collectionCode: Priv. Coll. J. Kahanpää; basisOfRecord: PreservedSpecimen
- **Occurrence:** recordedBy: Jukka Salmela; Jari Ilmonen; individualCount: 1; sex: male; **Taxon:** scientificName: Achalcus nigropunctatus; order: Diptera; family: Dolichopodidae; scientificNameAuthorship: Pollét & Brunhues, 1996; **Location:** country: Finland; stateProvince: PPp; municipality: Tervola; locality: Karhakkamaa; verbatimCoordinates: 7346879:3415620; verbatimCoordinateSystem: ykj; decimalLatitude: 66.203; decimalLongitude: 25.123; geodeticDatum: WGS84; coordinatePrecision: 200; **Identification:** identifiedBy: Jere Kahanpää; **Event:** samplingProtocol: Malaise trap; eventDate: 2004-06-28/08-02; **Record Level:** collectionCode: Priv. Coll. J. Kahanpää; basisOfRecord: PreservedSpecimen
- **Occurrence:** recordedBy: Jere Kahanpää; individualCount: 1; sex: male; **Taxon:** scientificName: Achalcus nigropunctatus; order: Diptera; family: Dolichopodidae; scientificNameAuthorship: Pollét & Brunhues, 1996; **Location:** country: Finland; stateProvince: KP; municipality: Kruunupyy; locality: Hällörsfjärden; verbatimCoordinates: 707486:329832; verbatimCoordinateSystem: ykj; decimalLatitude: 63.717; decimalLongitude: 22.914; geodeticDatum: WGS84; coordinatePrecision: 200; **Identification:** identifiedBy: Jere Kahanpää; **Event:** samplingProtocol: Malaise trap; eventDate: 2005-07-02/07-29; **Record Level:** collectionCode: Priv. Coll. J. Kahanpää; basisOfRecord: PreservedSpecimen

#### Biology

Locally common on open seashore or lakeshore wetlands characterised by *Potentilla palustris* intermixed with large *Carex* species. Unlike *Achalcus flavicollis* (Meigen, 1824) and *Achalcus vaillanti* Brunhes, 1987, *Achalcus nigropunctatus* is only rarely caught in pure *Phragmites* stands.

#### Distribution

New to Finland. Previously found in the United Kingdom, Germany, Switzerland, France, the Czech Republic and Slovakia (Drake 2008, Pollet 2009, Pollet 2013).

## Supplementary Material

XML Treatment for Microphorella praecox

XML Treatment for Hydrophorus callosoma

XML Treatment for Medetera belgica

XML Treatment for Medetera fumida

XML Treatment for Medetera prjachinae

XML Treatment for Medetera seguyi

XML Treatment for Medetera zinovjevi

XML Treatment for Dolichopus annulitarsis

XML Treatment for Dolichopus cilifemoratus

XML Treatment for Dolichopus costalis

XML Treatment for Dolichopus lancearius

XML Treatment for Dolichopus nigripes

XML Treatment for Sybistroma discipes

XML Treatment for Argyra setulipes

XML Treatment for Asyndetus latifrons

XML Treatment for Chrysotus cupreus

XML Treatment for Campsicnemus femoratus

XML Treatment for Micromorphus claripennis

XML Treatment for Achalcus nigropunctatus

## Figures and Tables

**Figure 1a.**
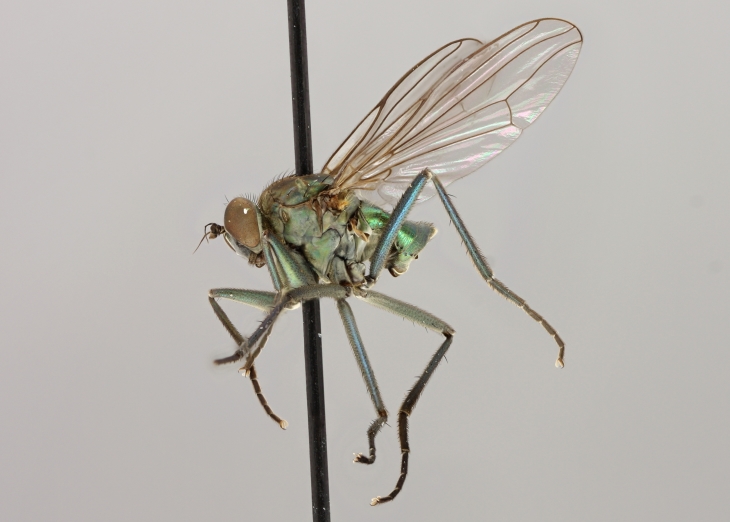
*Hydrophorus callosoma* Frey, male

**Figure 1b.**
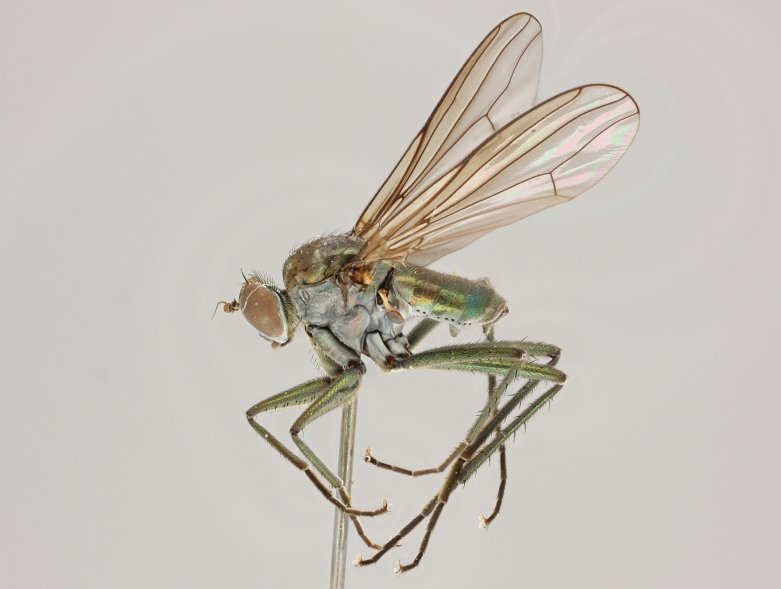
*Hydrophorus altivagus* Aldrich, male

**Figure 1c.**
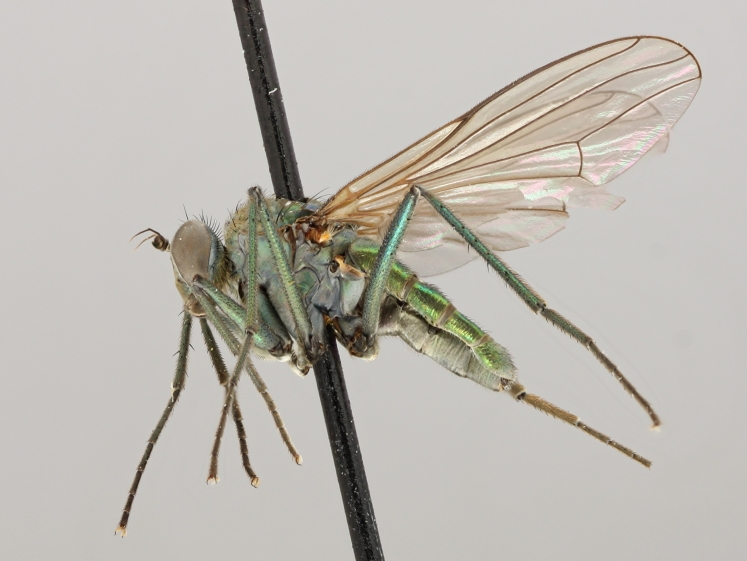
*Hydrophorus callosoma* Frey, female (lectotype of *Hydrophorus callosoma* Frey)

**Figure 1d.**
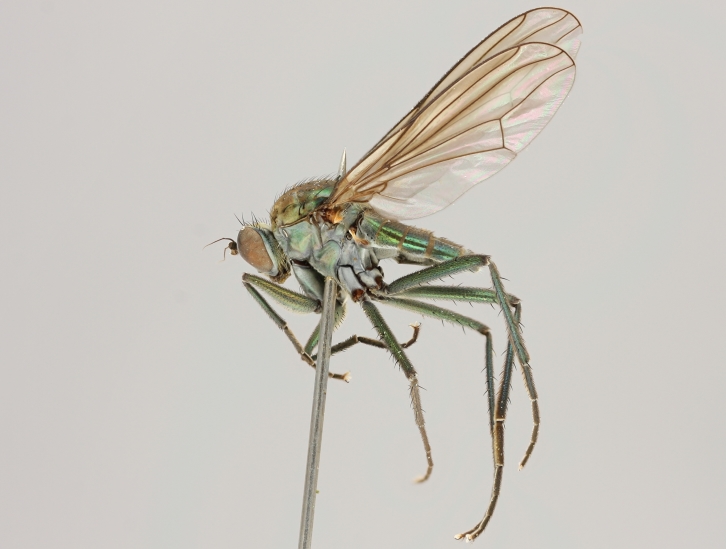
*Hydrophorus altivagus* Aldrich, female

**Figure 1e.**
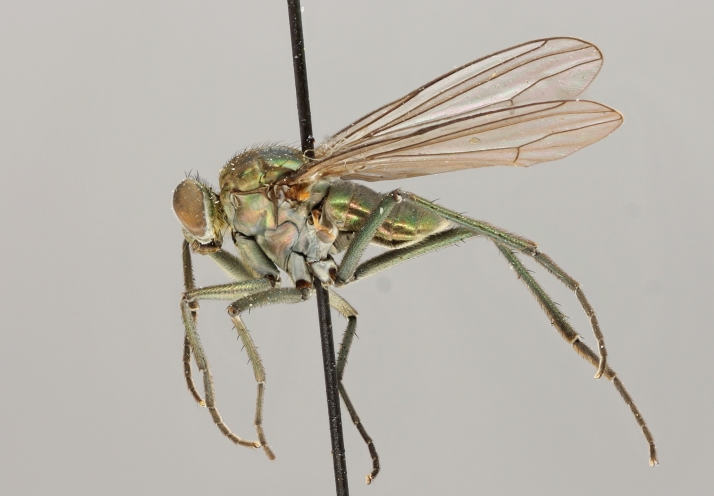
*Hydrophorus callosoma* Frey, female (paralectotype of *Hydrophorus albosignatus* Ringdahl)

**Figure 2a.**
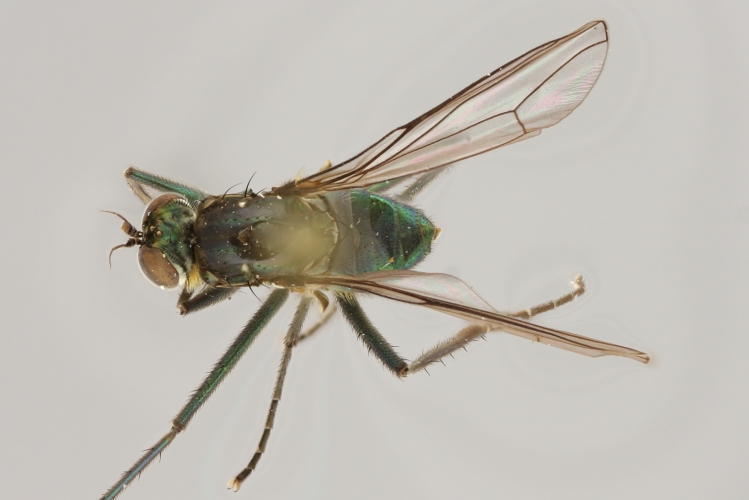
*Hydrophorus callosoma* male

**Figure 2b.**
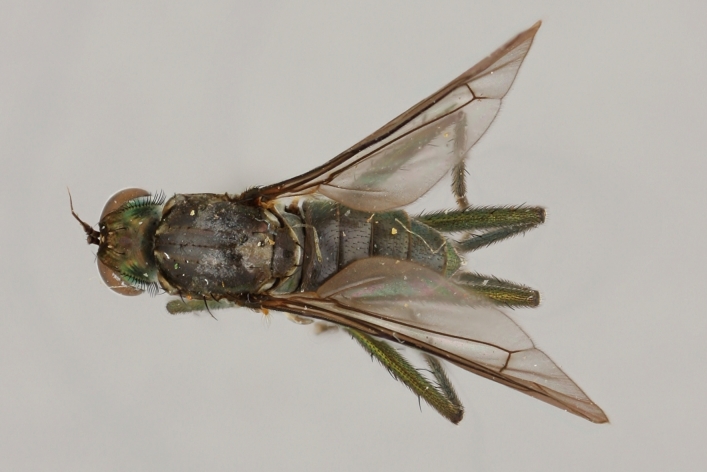
*Hydrophorus altivagus* male

**Figure 2c.**
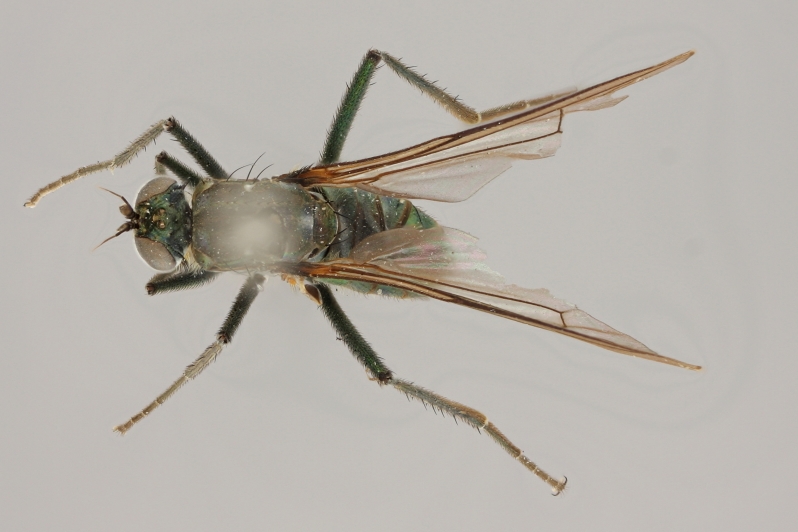
*Hydrophorus callosoma* female (lectotype of *Hydrophorus callosoma* Frey)

**Figure 2d.**
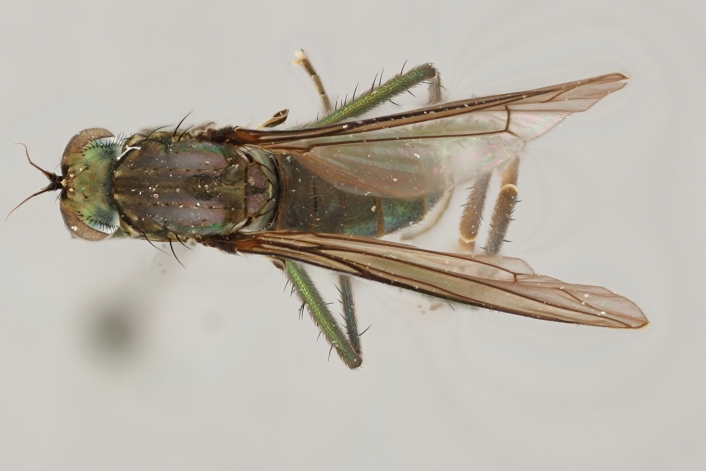
*Hydrophorus altivagus* female

**Figure 2e.**
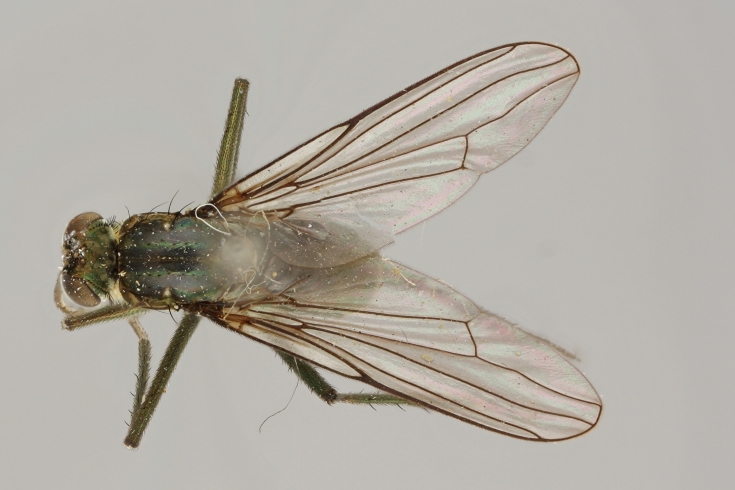
*Hydrophorus callosoma* female (paralectotype of *Hydrophorus albosignatus* Ringdahl)

**Figure 3a.**
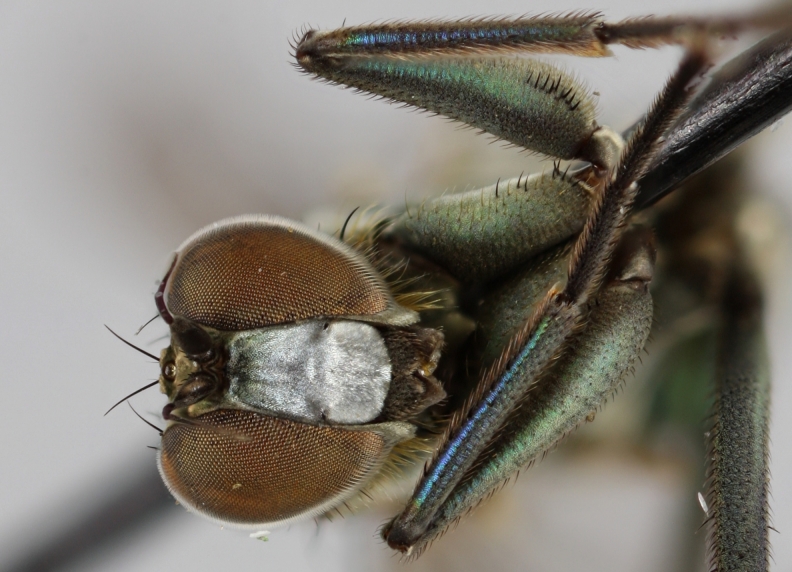
*Hydrophorus callosoma* male

**Figure 3b.**
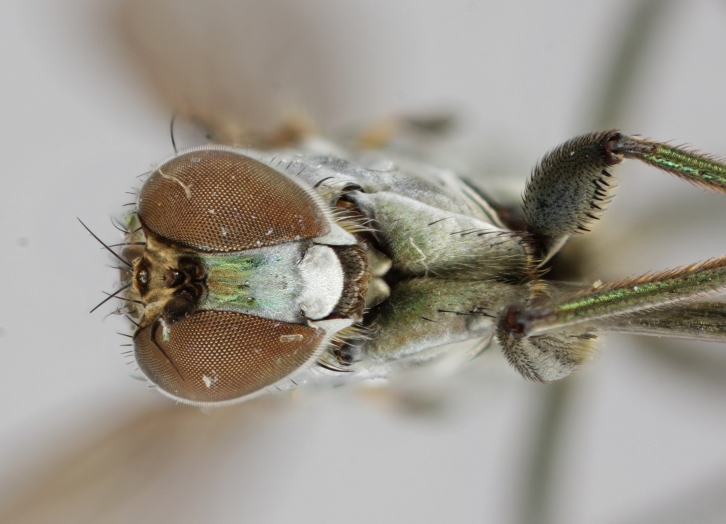
*Hydrophorus altivagus* male

**Figure 3c.**
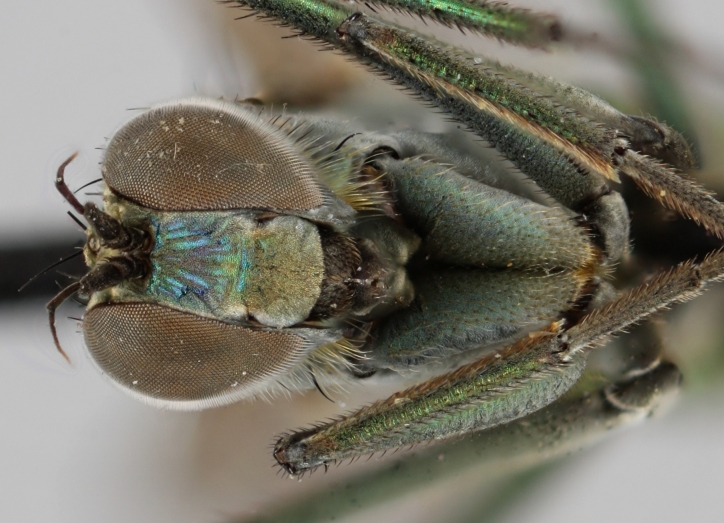
*Hydrophorus callosom*a female (lectotype of *Hydrophorus callosoma* Frey)

**Figure 3d.**
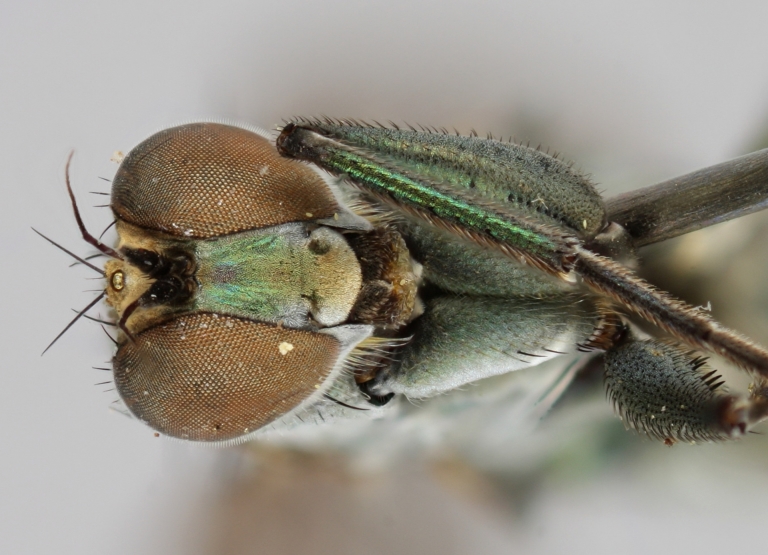
*Hydrophorus altivagus* female

**Figure 3e.**
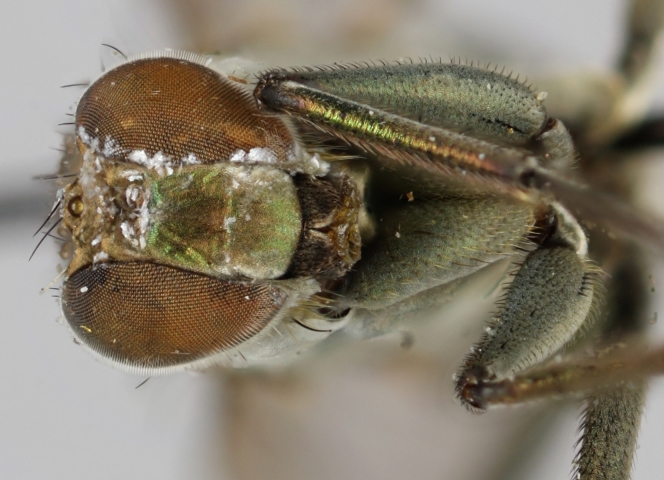
*Hydrophorus callosoma* female (paralectotype of *Hydrophorus albosignatus* Ringdahl)

**Table 1.** Character table for distinguishing *Hydrophorus callosoma* Frey from *Hydrophorus altivagus* Aldrich. See Figs 1, 2, 3 for illustrations of the characters.

character	*Hydrophorus callosoma* Frey	*Hydrophorus altivagus* Aldrich
face	wide, parallel-sided	narrower, broadened towards the clypeus
face, colour (males only)	silver, narrowly greenish under the antennae	green ground color obscured by silver only in the lower third-half
clypeus, shape	width of clypeus not much less than the height of face	width of clypeus clearly less than height of face
fore femur	one compact row of 6-13 ventral setae restricted to the basal third or half. Anteroventral setae missing or at lost 1-3 small, scattered ones	venrtal row with 3-8 sete in basal half. Anteroventral row of setae well developed, with 6-12 setae, reaching the apical third
wing color (females only)	wings almost transparent in both sexes	wing membrane faintly brownish esp. before vein M_1+2_
spot above notopleural depression	white, round or oval in dorsal view	variable, usually small, dark to yellow, linear along notopleural suture. Never white and round
abdomen (females only)	lateral hairs of tergites 5 at let partially white in dorsal view. Usually also tergites 3-4 with partially pale lateral hairs	lateral hairs (in dorsal view) on tergites 3-5 black. Note that the ventral hairs on tergites are usually white.
